# The ^1^H-NMR-based metabolite profile of acute alcohol consumption: A metabolomics intervention study

**DOI:** 10.1371/journal.pone.0196850

**Published:** 2018-05-10

**Authors:** Cindy Irwin, Mari van Reenen, Shayne Mason, Lodewyk J. Mienie, Ron A. Wevers, Johan A. Westerhuis, Carolus J. Reinecke

**Affiliations:** 1 Centre for Human Metabolomics, Faculty of Natural Sciences and Agriculture, North-West University (Potchefstroom Campus), Potchefstroom, South Africa; 2 Department of Statistics, Faculty of Natural Sciences and Agriculture, North-West University (Potchefstroom Campus), Potchefstroom, South Africa; 3 Department of Laboratory Medicine, Radboud University Nijmegen Medical Centre, Nijmegen, The Netherlands; 4 Biosystems Data Analysis, Swammerdam Institute for Life Sciences, University of Amsterdam, Amsterdam, The Netherlands; California State University Fresno, UNITED STATES

## Abstract

Metabolomics studies of disease conditions related to chronic alcohol consumption provide compelling evidence of several perturbed metabolic pathways underlying the pathophysiology of alcoholism. The objective of the present study was to utilize proton nuclear magnetic resonance (^1^H-NMR) spectroscopy metabolomics to study the holistic metabolic consequences of acute alcohol consumption in humans. The experimental design was a cross-over intervention study which included a number of substances to be consumed—alcohol, a nicotinamide adenine dinucleotide (NAD) supplement, and a benzoic acid-containing flavoured water vehicle. The experimental subjects—24 healthy, moderate-drinking young men—each provided six hourly-collected urine samples for analysis. Complete data sets were obtained from 20 of the subjects and used for data generation, analysis and interpretation. The results from the NMR approach produced complex spectral data, which could be resolved sufficiently through the application of a combination of univariate and multivariate methods of statistical analysis. The metabolite profiles resulting from acute alcohol consumption indicated that alcohol-induced NAD^+^ depletion, and the production of an excessive amount of reducing equivalents, greatly perturbed the hepatocyte redox homeostasis, resulting in essentially three major metabolic disturbances—up-regulated lactic acid metabolism, down-regulated purine catabolism and osmoregulation. Of these, the urinary excretion of the osmolyte sorbitol proved to be novel, and suggests hepatocyte swelling due to ethanol influx following acute alcohol consumption. Time-dependent metabolomics investigations, using designed interventions, provide a way of interpreting the variation induced by the different factors of a designed experiment, thereby also giving methodological significance to this study. The outcomes of this approach have the potential to significantly advance our understanding of the serious impact of the pathophysiological perturbations which arise from the consumption of a single, large dose of alcohol—a simulation of a widespread, and mostly naive, social practice.

## Introduction

The use of metabolomics in intervention, or challenge, studies greatly enhances the holistic understanding of the effects of single, or combined, consumed substances on metabolic pathways [1,2,3]. In this paper, we present the experimental design and outcome of an intervention study, which included a number of interventions: (1) vehicle only—commercial flavoured water containing sodium benzoate as preservative [3]; (2) a defined acute dose of alcohol, consumed alongside the vehicle; (3) an NAD supplement, taken one hour prior to the study, followed by consumption of the vehicle; and (4) the NAD supplement, followed by consumption of the vehicle and the alcohol dose. Alcohol oxidation by alcohol dehydrogenase (ADH) and aldehyde dehydrogenase (ALDH) results in the reduction of oxidized NAD (NAD^+^) to its reduced form (NADH), thereby generating a highly reduced cytosolic environment in hepatocytes. An increased NADH:NAD^+^ ratio has been described to influence several metabolic processes, resulting in, amongst others, decreased glycolysis [4], reduced Krebs cycle [5,6], and decreased gluconeogenesis [6,7]. The use here of NAD as a supplement is based on a perceived view that it has a therapeutic capacity if taken prior to alcohol consumption; the theory being that it supplements alcohol-induced NAD^+^ depletion.

The data set of the present study was generated from longitudinal (urine samples, collected hourly over a five-hour period), multi-subject (several experimental participants) and multi-group (in this case four, separate interventions) measurements, and expressed as multivariate data—numerous variables generated and identified through a metabolomics approach utilizing ^1^H-NMR spectroscopy. The main aim of the investigation was to determine the effect of acute (a single dose indigested in a short time) alcohol consumption, as well as the potential effect of an NAD supplement on the resulting metabolite profile. Several metabolomics studies have been reported on the pathophysiological consequences of chronic consumption and alcoholism in humans [8,9,10,11], as well as on chronic intragastric alcohol feeding of rodents, followed by mass spectrometric based metabolite profiling [12,13]. The metabolomics technique has been applied for global metabolite profiling using GC–MS [14,15], in several LC–MS studies [12,13,16,17], as well as in untargeted studies using ^1^H-NMR spectroscopy (examples hereof are summarized in Table 1). ^1^H-NMR spectroscopy is a robust method with broad metabolite-class coverage (albeit with limited sensitivity), and is well suited for the diverse metabolomics studies listed in Table 1 using rodents [18,19,20,21,22], selected human volunteers at risk for cardiovascular diseases [23], and population-based cohorts [24]. With the exception of the study by Bradford *et al*. [19], all the studies listed in Table 1 used serum or tissue material as the samples for the generation of the metabolite data. Here we report on an untargeted NMR metabolomics intervention study on acute alcohol consumption, which expands the limited data on alcohol-induced metabolic profiling in humans. This study followed a non-invasive sampling procedure (hourly-collected urine samples) which is ideal for generating the untargeted ^1^H-NMR-based metabolic profiles required for the longitudinal experimental design. As a biological waste material, urine is the biofluid of choice [25] to reflect the metabolic breakdown products following acute alcohol consumption, as well as any potential by-products derived from the gut microbiome, which has been shown to be important in several alcohol-related studies [14,16,26]. The data pre-processing applied to the spectral data in the present study are comparable to the approaches applied in the studies listed in Table 1, albeit with minor differences.

**Table 1:** A summary of the experimental designs, data analysis approaches and main metabolic conclusions of some NMR-based ethanol administration studies.

Authors (year) & Summary of design	Data pre-processing	Statistical analysis	Conclusion on metabolic observations
**Nicolas *et al* (2008)** **C**: Sprague–Dawley rats (N = 5). **S**: Serum and tissue. **E**: Single dose/binge protocol. **A**: Untargeted	(1) Zero-filled. (2) Fourier transformed. (3) Baseline corrected	**D**: Spectral bins. **T**: Mean centred. **G**: Pre vs Post. **U**: t-test. **M**: PCA	A cycle of aborted gluconeogenesis stimulated by the increased NADH/NAD ratio but short-circuited by decreased alanine levels during hepatic ethanol metabolism.
**Bradford *et al* (2008)** **C**: Male C57Bl/6J mice (N = 4). **S**: Urine, serum and liver. **E**: Chronic: 7–36 g/kg/day; Fed *ad libitum*. **A**: Untargeted	(1) Spectra phased. (2) Baseline corrected. (3) Normalized to the sum of all integrals set to 1000	**D**: Spectral bins. **T**: Pareto scaled. **G**: Pre vs Post. **U**: t-test. **M**: PCA & OPLS	Energy utilization is important in understanding the pathogenesis of alcohol induced liver injury, with acetylglutamine, n-acetylglycine and taurine potential novel non-invasive markers of alcohol consumption and oxidative stress.
**Masuo *et al* (2009)** **C**: Female Fisher rats (N = 5). **S**: Liver and brain. **E**: Chronic: 10 mo 15% Sake; Fed *ad libitum*. **A:** Untargeted	(1) Spectra normalize to total integral area. (2) Referenced to Na-TMS-tetra-1H-propionate	**D**: Spectral bins. **T**: Pareto scaled. **G**: Pre vs Post. **U**: t-test. **M**: PCA & PLS–DA	In liver: An attenuation of mitochondrial function; In brain: (1) Perturbed amino acids (Increased and decreased); and (2) Decreased N-acetyl-aspartate, taurine and GABA. Chronic Sake intake may cause alterations in the intoxicated body but also in the next generation.
**Fernando *et al* (2010)** **C**: Male Fischer rats (N = 344). **S**: Plasma and liver. **E**: Chronic: 5% alcohol diet. **A**: Lipidome	(1) Spectra manually phased. Referenced to TMS. (2) Spectra divided into equal bins of 0.01 ppm width. (3) Baseline corrected	**D**: Spectral bins. **T**: Auto scaled. **G**: Pre vs Post. **U**: t-test. **M**: Hierarchical clustering & PCA	Alcohol consumption alters metabolism of cholesterol, triglycerides and phospholipids that could contribute to the development of fatty liver and indicates that fatty liver precedes oxidative stress and inflammation.
**Yoseph *et al* (2013)** **C**: Male FVB/N mice (N: 8–9). **S**: Pancreatic tissue. **E**: Chronic; Fed *ad libitum* with increased interventions. **A**: Untargeted/Focus: Pancreatic metabolome	(1) Spectra phased. (2) Baseline corrected. (3) Spiking experiments to validate suspected metabolites identified in the spectra.	**D**: 52 Metabolites (mM). **T**: Variance scaled. **G**: Pre vs Post. **U**: t-test & MW test. **M**: PLS–DA & One-way ANOVA (Tukey post-test)	Pancreatic metabolome following chronic alcohol intake indicates increased acetate, adenosine, xanthine, acetoacetate, 3-hydroxybutyrate and betaine; and (2) decreased cytidine, uracil, fumarate, creatine phosphate creatine, and choline. Mice with chronic alcohol ingestion have increased mortality when encountered with sepsis.
**Vázquez-Fresno *et al* (2012)** **C**: Males volunteers at risk (Age≥55; N = 61). **S**: 24-hour urine. **E**: Nutritional wine. **A:** Untargeted and selected metabolites	*Untargeted*: (1) Spectra divided into equal bins. (2) Profiling integration. (3) Spectra phased. (4) Baseline corrected. (5) Calibrated. *Selected metabolites*: Chenomx NMR Suite 7.0 profiler compared on: HMDB/BMRB/MMCD and literature	**D**: Spectral bins. **T**: Pareto scaled. **G**: Pre vs Post. **U**: t-test; Pearson’s correlation. **M**: ANOVA & Fisher’s LSD	(1) Food metabolome: Mannitol (diet); tartrate (wine intake). (2) Endogenous modifications after wine consumption indicated by branch-chain amino acids. (3) Gut metabolites, 4-hydroxy phenylacetate and hippurate
**Wurtz *et al* (2016)** **C**: 3 population-based cohorts, (meta-analysis N = 9778). **S**: Serum. **E**: Habitual consumption. **A**: Targeted lipids and 86 metabolic measures	Automated NMR metabolite profiling: Robotics-controlled and fully automated with a capacity of about 150–180 samples in 24 h. Integrated computational methods for the data-driven systems biology approach to biomedical research	**D**: Computational generated. **T**: Log_e_ transformed & SD Scaled. **G**: Cross-sectional (i.e. 2 group comparisons). **U**: Bonferroni method; t-test & MW test. **M**: Linear regression (R-squared)	Prominent metabolic associations with alcohol consumption include monounsaturated fatty acids, omega-6 fatty acids, glutamine, citrate and lipoprotein particle size. Many of these cardiometabolic biomarkers strongly associated with alcohol intake as HDL cholesterol.
**Irwin *et al* (the present study)** **C**: Healthy males (Age~22; N = 24). **S**: Hourly urine (5 times). **E**: Acute (1.5 g vodka/kg). **A:** Untargeted and quantified metabolites	Normalized relative to the creatinine at 4.05 ppm. Baseline corrected– 50% zero-filter. Batch comparisons using QC samples	**D**: Spectral bins (excluding H_2_O region). **T**: log_e_ transformed; Auto-scaled. **G**: Cross-over, time dependent matched series. **U**: WR-test; ES; FC. **M**: Hierarchal cluster; Multi-level PCA. The NAD-effect: 2-way RM ANOVA	Aims: (1) Effect of vehicle consumption: Irwin *et al* (2016) PLOS ONE. 11:e0167309. (2) Impact of consumption of a single large dose of alcohol (time dependence). (3) Statistical analysis of quantified metabolites following consumption. (4) Effect of NAD supplementation. (5) Model of the metabolite profile following acute alcohol consumption

**Abbreviations used in columns 1 and 3:** C: Cases studied (experimental animals or humans); S: Samples used for metabolite identification; E: Ethanol exposure; A: Approach for the generation of the variable or metabolite data; N: number of experimental cases studied; D: Data used for statistical analyses; T: Transformation and scaling approach; G: Groups compared; U: Univariate analyses applied; M: Multivariate analyses applied.

**Other abbreviations**: ES: Effect Size; FC: Fold Change; ANOVA: Analysis of variance; MW: Mann-Whitney test; PCA: Principal Component Analysis; PLS-DA: Partial Least Squares-Discriminant Analysis; RM: Repeated Measures; WC: Wilcoxon test.

The statistical methods used in the intervention studies listed in Table 1 all use a pre *vs* post approach—multivariate model-based analysis (PCA, PLS–DA, OPLS and ANOVAs) of the data was performed to determine the metabolites responsible for the separation of the control (“pre”) versus the alcohol-treated (“post”) groups. Analysis of the data set from the present study is theoretically complex and required methods specifically designed for longitudinal, multi-subject, multi-group and multivariate data [27,28]. We accordingly generated a matched-sample series through a cross-over study of participating subjects, collecting samples over a distinct time frame. First, data from the “vehicle only” and “alcohol plus vehicle” interventions were compared at each time point to determine the impact of the consumption of a single, large dose of alcohol. Next, the statistical analysis focused on the metabolic perturbations due to the acute consumption of alcohol, measured over time. A shortlist of significantly perturbed NMR spectral bins was compiled and quantified as relative concentrations for biological interpretation. Finally, the effect of NAD supplementation was evaluated for a select list of biologically relevant metabolites.

Most remarkable in the present study was the highly increased presence of urinary sorbitol, a response to alcohol consumption not observed in the studies listed in Table 1, nor previously reported elsewhere. The other main metabolic observations that emanated from this NMR-based intervention study was the increased levels of urinary lactic acid (indicated as a biomarker in two of the listed rodent studies) and hypoxanthine (not observed in the studies listed in Table 1, but observed in another metabolomics study on patients with liver cirrhosis [11]). Both these observations strongly support the view that the reduced cytosolic environment is the primary metabolic consequence of acute alcohol consumption. Taken together, the metabolic information acquired from this metabolomics study further underscores the view that alcohol consumption is associated with severe risks and remains one of the world’s leading health risk factors for disability, morbidity and mortality. This is succinctly expressed in the 2014 World Health Organization global status report on alcohol [29]: “*Of all deaths worldwide*, *5*.*9% are attributable to alcohol consumption; this is greater than*, *for example*, *the proportion of deaths from HIV/AIDS (2*.*8%)*, *violence (0*.*9%) or tuberculosis (1*.*7%)”*.

## Materials and methods

### Chemicals and reagents

The substances used for the interventions reported here were: commercial flavoured water as vehicle (aQuellé lemon-flavoured sparkling water, containing carbonated natural spring water; fructose; citric acid; flavouring; sodium benzoate preservative; sodium cyclamate, aspartame, acesulfame K non-nutritive sweeteners; and vitamin C– www.aquelle.co.za, product of South Africa); commercial alcohol for consumption (Smirnoff No. 21 triple-distilled vodka: 43% alcohol–product of South Africa); NAD (*NAD ASSIST*–product of Future Health, South Africa); and commercial bottled water (Valpré still spring water, inorganic contents specified–product of South Africa). The internal standard for the ^1^H-NMR analysis was trimethyl-2,2,3,3-tetradeuteropropionic acid (TSP, sodium salt; Sigma Aldrich).

### Experimental subjects and protocol

The participants in the intervention study consisted of a group of 24 medically confirmed healthy males, of various ethnicities, between 20 and 24 years of age. All of the participants were in a healthy, athletic condition, with weights in the 61–92 kg range. They were neither alcohol addicts nor total abstainers, but confirmed their use of alcohol at a moderate social level. No participants took any medication, all were asked to refrain from vitamins, minerals, and other supplementation, and were requested to follow a similar dietary and lifestyle pattern for the duration of the study. The complete protocol, described previously [3], was approved by the Health Sciences Ethical Committee of North-West University (Ethical approval number: NWU-00045-12-S1) and conducted in accordance with guidelines for good clinical practice. The study was performed at the Health Clinic of the university, under the supervision of a medical doctor as well as a nurse, and all participants could leave the premises only after approval by the doctor.

The experiments were conducted on Saturday mornings between 08:00 and 12:00. All participants had to abstain from breakfast and had to provide an early morning urine sample, collected one hour before the start of the experiment (time –1). The participants were randomly assigned to an intervention group until all 24 had participated in all four interventions (“vehicle only”, “alcohol plus vehicle”, “vehicle plus NAD” and “vehicle plus alcohol and NAD”). Owing to commitments of some participants, the experiments were performed over a period of 7 consecutive Saturdays. However, three of the participants failed to participate in all four interventions, and their samples were therefore excluded from the data generation process. Through data analysis, one further participant was marked as an outlier in one of the interventions, and was therefore also excluded from further analyses. Thus, the data generated were based on the complete sets of information obtained from 20 participants.

The four interventions consisted of the consumption of: (1) 500 mL lemon-flavoured water only, to measure the baseline effect of the vehicle; (2) 1.5 g vodka per kg body mass; (3) one tablet containing 50 mg NAD; and (4) 1.5 g vodka per kg body mass plus 50 mg NAD. In all three of the latter interventions, the substances were consumed with 500 mL flavoured water as vehicle. NAD (where applicable) was taken one hour before the start of the experiment, directly after collection of the first (time –1) urine sample. The dose and time schedule for consuming the alcohol (maximally five minutes) comply with established criteria to result in a tolerable but moderately severe level of acute alcohol intoxication [30]. All participants were provided with 1.5 L pure spring water, which was the only substance that could be consumed over the four-hour period of sample collection. Urine samples were collected at time 0, just prior to consumption of the substances, followed by four further samples at 1, 2, 3 and 4 hours thereafter, providing six samples in total from each participant for each of the four interventions. All samples were treated, stored, prepared and analysed according to the protocol described previously [3] and included in Sections A.1 and A.2 in S1 File. Although repeatability and reproducibility are not major concerns in NMR analyses [31], the measurement design included the use of pooled quality control (QC) samples to estimate any batch effect or other interfering analytical aspect. The collected samples were analyzed in 24 separate batches, each batch containing the 24 samples of a single subject and three QC samples. The batch analysis order is given in Section A.1 in S1 File. The quality assurance results (given in Section A.3.2 and Figure A in S1 File) indicated that the QC samples clustered close together and therefore no batch correction was required.

Uric acid does not have NMR-detectable protons at physiological pH, making it essentially ‘‘NMR invisible” [25]. Uric acid concentrations were therefore determined in each sample individually on a ThermoFisher Scientific Clinica Chemistry Analyzer (type 863), using ThermoFisher Scientific Uric Acid Reagents (details on the analysis are included in Section B in S1 File).

### Data analysis

The original NMR spectral data (referred to in Section C in S1 File and available in S2 File) were subjected to pre-processing—data were normalized relative to the CH_2_ creatinine peak (at 4.05 ppm), and very low values were replaced with zero, before performing a 50% zero-filter (details in Section A.3.1 in S1 File). Further bin reduction, based on batch comparisons of the coefficient of variation of the bins from the QC samples, yielded 289 bins for further analyses. The resulting data were analysed along all dimensions in order to understand the metabolic effect of the different interventions in time, as well as the differences between the participants, even when considering a relatively homogeneous group such as the subjects involved in this study. The analysis was performed on different cross-sections and blocks of the data tensor. Univariate as well as multivariate methods were applied. Data were log transformed (natural log with shift parameter equal to 1) and auto-scaled (per bin) prior to univariate (parametric tests only) and multivariate analysis.

The first analysis aimed to shed light on the impact of the consumption of a single, large dose of alcohol. To illustrate this effect, data from the “vehicle only” and “alcohol plus vehicle” interventions were compared at each point in time. Cross-sectional analysis made use of multivariate techniques, including hierarchal cluster analysis (Euclidian distance and Ward linkage) and multi-level principal component analysis (ML–PCA) [32]. Univariate approaches included determining Wilcoxon signed-rank test (WRT) p-values and associated effect size, as well as fold change (FC) values.

Next, statistical analysis focused on the metabolic perturbations that result from the consumption of alcohol as measured in time. Through the application of a combination of statistical methods, and using time 0 as a reference point, or control group, a shortlist of significantly perturbed NMR spectral bins was compiled and quantified from the “alcohol plus vehicle” intervention data for biological interpretation. This list of important bins was extended through the inclusion of metabolites that were well presented in the spectra, although they did not contribute significantly to the statistical differentiation within the “alcohol plus vehicle” consumption profiles.

Lastly, the effect of NAD supplementation was of interest. In order to evaluate this intervention, a select list of biologically relevant metabolites was modelled across the time period and across all four interventions using a two-way repeated measures analysis of variance (2-way RM ANOVA) model. The significance of the differences was calculated using Greenhouse–Geisser-corrected p-values. In addition, Wilcoxon signed-rank tests were performed to calculate the significance of the differences between selected pairs of measurements.

## Results

**Fig 1:**
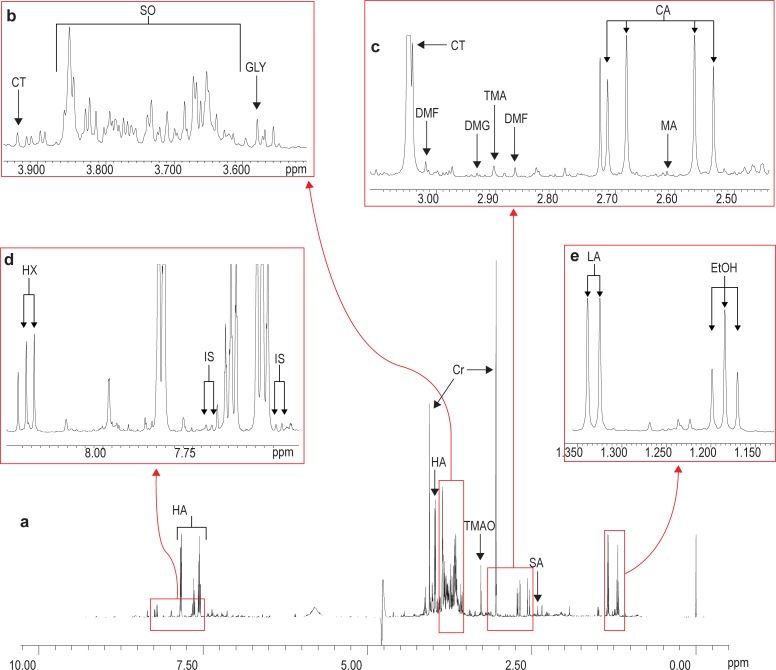
Representative ^1^H-NMR spectrum of urine collected one hour following the “alcohol plus vehicle” intervention. EtOH = ethanol (1.18 t, 3.64 q); LA = lactic acid (1.33 d, 4.12 q); SA = succinic acid (2.41 s); TMAO = trimethylamine-N-oxide (3.27 s); CA = citric acid (2.61 AB); MA = methylamine (2.61 s); TMA = trimethylamine (2.90 s); DMG = N,N-dimethylglycine (2.93 s); DMF = N,N-dimethylformamide (2.87 s, 3.02 s); CT = creatine (3.04 s, 3.93 s); GLY = glycine (3.57 s); SO = sorbitol (3.60–3.69 m, 3.73 d, 3.74–3.80 m, 3.82 d, 3.85 m); IS = indoxyl sulphate (7.51 d, 7.70 d); HX = hypoxanthine (8.20 d); HA = hippuric acid (3.97 d, 7.56 tt, 7.64 tt, 7.84 dd); Cr = creatinine (3.05 s, 4.06 s). [Not observed in the present spectrum: fumaric acid (6.52 s) and 3-hydroxybutyric acid (1.20 d, 2.36 m, 4.15 m)].

An example of a ^1^H-NMR spectrum (10.00 to 0.00 ppm), generated from the analysis of a representative experimental sample collected one hour following the “alcohol plus vehicle” intervention, is shown in Fig 1A, together with enlargements of four sections of the spectrum to further illustrate some detail (Fig 1B–1E).

**Fig 2:**
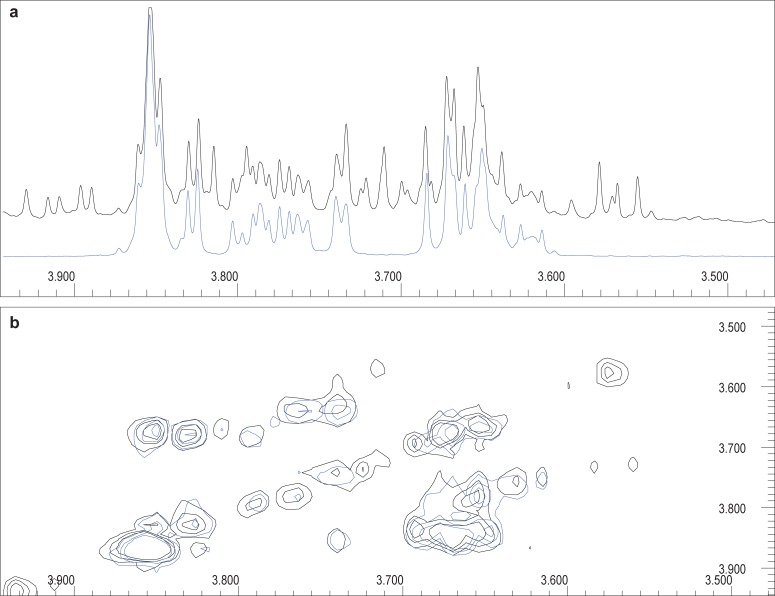
Confirmation of sorbitol annotation. (a) 1D ^1^H-NMR of a representative urine sample collected one hour after alcohol consumption, zoomed into the 3.50–3.90 ppm region (black), compared to the pure compound spectrum of sorbitol (blue). (b) Correlating 2D ^1^H-NMR COSY, confirming the sorbitol annotation based upon proton correlation.

The dominating peaks in Fig 1A were from ethanol (due to the alcohol consumption), hippuric acid (the biotransformation product of benzoic acid, derived from the gut microbiome and present in the vehicle used in all the interventions, as well as observed in a moderate red wine nutritional study) [23], creatinine (a normal constituent of urine), and trimethylamine-N-oxide (TMAO), a known osmolyte and protein stabilizer. Most notable was the presence of an exceedingly complex area, approximately between 3.60 and 3.90 ppm (Fig 1B). Interpretation of this region is particularly difficult, since it may contain overlapping resonances from several metabolites. The strong signals observed at 3.60–3.69, 3.73, 3.74–3.80 and 3.85 ppm were suggestive of sorbitol in the urine, following alcohol consumption. This suggestion was confirmed by comparing the spectra obtained through two-dimensional (2D) correlation spectroscopy (COSY) analysis of an experimental sample, obtained one hour after alcohol consumption, and a sample containing sorbitol as a standard (Fig 2).

Next, an area with several related amines or amine derivatives is shown in Fig 1C, while Fig 1D indicates the presence of hypoxanthine (an intermediate in the catabolism of purines towards uric acid) and indoxyl sulphate (a biotransformation product of tryptophan). Finally, Fig 1E indicates the presence of ethanol itself, as well as the lactic acid doublet (1.33 ppm), the main known marker of lactic acidosis, and one of the primary effects of acute alcohol consumption.

**Fig 3:**
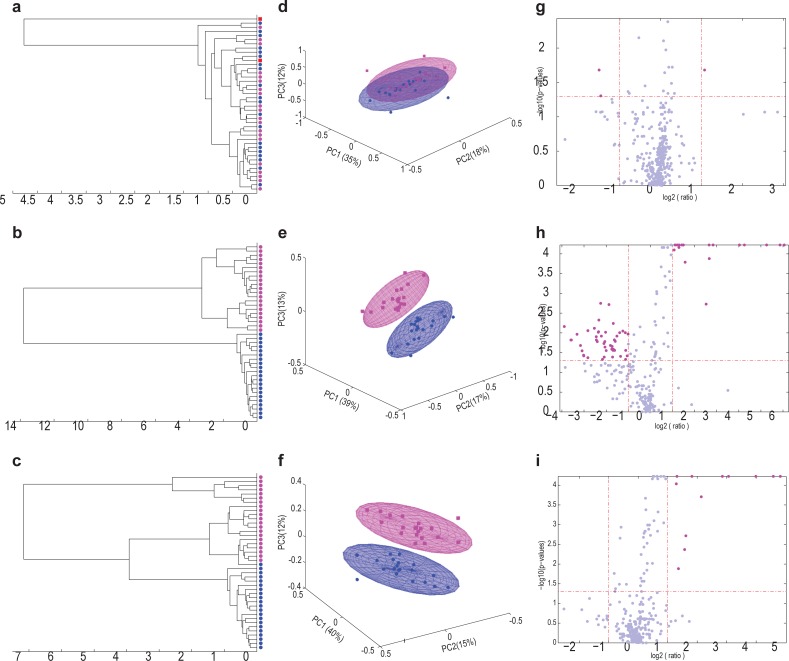
Group separation between participants, based on equidistant binned spectral data from the “vehicle only” and the “alcohol plus vehicle” interventions, illustrated as dendrograms, ML–PCA plots and Volcano plots. The respective analyses were constructed on subsets of the data representing the same three time points—time 0 (a, d and g), 2 hours (b, e and h) and 4 hours (c, f and i) following the two interventions. Data from the 21 participants in the dendrograms and ML–PCA plots are shown as blue dots/areas for the “vehicle only” intervention and pink dots/areas for the “alcohol plus vehicle” intervention. The single outlier is shown as a red square in the dendrograms. All data from this participant were excluded from further analyses, resulting in the analysis of the data from a total of 20 participants.

Spectral analysis of the representative sample gives a clear indication of the important metabolites present in urine following alcohol consumption (Fig 1), but no information on the underlying dynamic effects following the consumption. The next step was therefore to determine the time-dependent impact of the consumption of a single, large dose of alcohol. Statistical analyses of the data obtained from the experimental samples from the “vehicle only” and “alcohol plus vehicle” interventions provide such information, and are illustrated in Fig 3. The NMR spectra generated were dominated by the presence of hippuric acid (see [3]). Exclusion of hippuric acid from the data set, however, did not significantly affect the relevant information on alcohol consumption.

Fig 3A and 3D (time 0) showed no group separation prior to vehicle or alcohol consumption. It did, however, become apparent that one individual may have used an exogenous substance on the day of his “vehicle only” intervention experiment, which completely separated this person from all others in the group (indicated as a red square in Fig 3A). This rendered the data from this individual unfit for analysis, producing the final number of 20 cases used for further analysis. The bin profiles obtained two hours after the “vehicle only” and “alcohol plus vehicle” interventions clearly indicated group differences in the unsupervised analyses due to the addition of alcohol (Fig 3B and 3E). The Volcano plot from data collected one hour after alcohol consumption revealed a larger number of bins with significant up- and down-regulated values (p ≤ 0.05 and │FC│≥ 2) relative to the number of bins from data collected one hour after vehicle consumption (data not shown). This number of significant bins increased to at least 58 out of the 289 bins (20%) two hours following consumption (Fig 3H). The number of these bins progressively decreased 3 and 4 hours after consumption, becoming only 13 bins after 4 hours that differed significantly between the two interventions (Fig 3I).

Given this marked effect, the next set of analyses were performed to identify and rank the bins most affected by the consumption of alcohol using time 0 as a point of reference. Bins were shortlisted if they differed significantly within the “alcohol plus vehicle” intervention at any point in time (relative to time 0) based on a significant WRT p-value ≤ 0.05 and |FC| ≥ 2. This shortlist of bins was linked to a set of metabolites and quantified. This list, including only the most perturbed metabolites, was then extended through the inclusion of metabolites which were well presented in the spectra, but did not contribute significantly to the statistical differentiation within the “alcohol plus vehicle” consumption profiles. Quantified uric acid data (not identified by NMR analysis, but determined individually for each urine sample) was also added to this final shortlist of 13 important metabolites. The concentrations of these 14 metabolites, at all five time points related to the “alcohol plus vehicle” intervention, are given in Table 2, together with the relevant summary statistics, determined one (early effect) and four (late effect) hours after alcohol consumption. Time –1 is not included in Table 2 since it reflects the past several hours prior to the intervention, and is not related to the “alcohol plus vehicle” intake. The purpose of Table 2 was then to rank metabolites for biological interpretation of the consequences of the intervention.

**Table 2:** Quantified data of important metabolites following alcohol consumption.

Variable	Time 0 vs Time 1 (early effect)		Time 0	Time 1	Time 2	Time 3	Time 4	Time 0 vs Time 4 (late effect)	
	WRT p-value	WRT Effect Size	Mean	Mean	Mean	Mean	Mean	WRT p-value	WRT Effect Size
	[BH Adjusted p-value]	[FC]	[SD]	[SD]	[SD]	[SD]	[SD]	[BH Adjusted p-value]	[FC]
DMF (N,N-dimethylformamide)	0.057	0.301	15.98	16.58	15.64	16.01	17.12	0.017	0.378
	[0.079]	[+1.038]	[2.292]	[2.613]	[2.795]	[2.308]	[2.699]	[0.047]	[+1.071]
DMG (N,N-dimethylglycine)	0.052	0.307	8.472	9.656	9.190	8.168	8.438	1.000	0.000
	[0.079]	[+1.140]	[7.965]	[10.02]	[9.230]	[7.640]	[7.274]	[1.000]	[–1.004]
Ethanol	<0.0001	0.620	0.000	1389	6448	3440	1912	<0.0001	0.620
	[0.0004]	[> +100]	[0.000]	[1556]	[2673]	[1984]	[1250]	[0.001]	[> +100]
Glycine	0.044	0.319	160.2	183.4	151.4	146.6	158.3	0.601	0.083
	[0.077]	[+1.145]	[84.90]	[86.26]	[44.68]	[50.45]	[56.11]	[0.701]	[–1.012]
Hippuric acid	0.313	0.159	220.4	380.2	87.66	138.1	178.9	0.100	0.260
	[0.313]	[+1.725]	[217.2]	[393.7]	[124.4]	[344.7]	[333.7]	[0.156]	[–1.232]
Hypoxanthine	<0.0001	0.620	9.604	78.63	29.82	14.44	12.48	0.044	0.319
	[0.0004]	[+8.187]	[7.029]	[45.97]	[14.99]	[5.716]	[5.191]	[0.077]	[+1.299]
Indoxyl sulphate	0.002	0.490	24.30	27.86	24.87	21.51	22.87	0.794	0.041
	[0.005]	[+1.147]	[10.29]	[13.42]	[16.04]	[14.25]	[11.19]	[0.855]	[–1.063]
Lactic acid	0.001	0.508	70.90	179.9	92.74	75.96	72.79	0.006	0.431
	[0.004]	[+2.537]	[61.39]	[274.8]	[19.59]	[14.04]	[16.26]	[0.022]	[+1.027]
Methylamine	0.006	0.431	4.556	5.282	5.937	5.186	5.059	0.033	0.336
	[0.013]	[+1.159]	[2.146]	[2.477]	[2.256]	[2.380]	[2.263]	[0.073]	[+1.110]
Sorbitol	<0.0001	0.620	0.000	653.2	852.8	426.2	289.0	0.000	0.614
	[0.0004]	[> +100]	[0.000]	[462.0]	[625.7]	[331.6]	[253.3]	[0.001]	[> +100]
Taurine	0.062	0.295	100.6	108.2	109.7	113.5	118.7	0.006	0.437
	[0.079]	[+1.076]	[29.71]	[32.54]	[29.86]	[37.45]	[36.49]	[0.022]	[+1.180]
TMAO (trimethylamine N-oxide)	0.002	0.502	51.18	58.32	60.31	62.45	62.00	0.037	0.331
	[0.004]	[+1.139]	[23.90]	[25.48]	[29.26]	[35.68]	[32.48]	[0.073]	[+1.211]
Trimethylamine	0.100	0.260	1.753	1.987	1.763	1.654	1.615	0.167	0.218
	[0.117]	[+1.133]	[1.229]	[1.657]	[1.680]	[1.578]	[1.436]	[0.234]	[–1.085]
Uric acid	0.247	0.183	0.963	0.713	0.336	0.487	0.743	0.211	0.198
	[0.266]	[–1.351]	[0.760]	[0.279]	[0.168]	[0.243]	[0.566]	[0.269]	[–1.296]

All quantified values, except those for uric acid (expressed as mmol/L), are from NMR-determined urine analyses, and are expressed as μmol metabolite/mmol creatinine. WRT p-values are based on the comparison of the respective metabolite concentrations relative to time 0 for time 1 and time 4 of the “alcohol plus vehicle” intervention. P-values adjusted for multiple testing (14 tests in total) are also reported based on the Benjamini & Hochberg (BH) approach for controlling the rate of false discoveries. Positive and negative fold change values indicate up- and down-regulation of metabolites, relative to time 0, respectively.

The data summarized in Table 2 indicate that seven metabolites (ethanol, hypoxanthine, indoxyl sulphate, lactic acid, methylamine, sorbitol and TMAO) were significantly up-regulated (p ≤ 0.05) in the first hour following alcohol consumption. Five of these metabolites (ethanol, hypoxanthine, lactic acid, sorbitol and TMAO) remained up-regulated at every time point up to time 4 after alcohol consumption, although less significantly so (p ≤ 0.05) than after the first hour. This observation illustrates a general characteristic of metabolic profiling, well known in the area of inherited diseases: serial examinations of urinary metabolites show that the amounts of these acids excreted varies greatly in time following a perturbation, mostly due to different metabolic consequences related to the perturbation. The uric acid excretion profile observed in this study further emphasizes this characteristic. The profile of urinary uric acid differed distinctly from those of the other metabolites mentioned—its concentration decreased from time 0, and became significantly reduced 2 and 3 hours after alcohol consumption (p ≤ 0.05 relative to time 0). Thereafter, its concentration steadily increased to near its initial level after 4 hours. Three other metabolites, DMF, taurine and glycine, became significantly up-regulated at some stage following alcohol consumption. The up-regulation of two metabolites, DMG and trimethylamine, was not significant at any time point relative to time 0 following the intervention. The excretion profile of hippuric acid followed the same trend as previously reported for the “vehicle only” intervention [3].

These mean values of the metabolites over time indicate the dynamic aspect following alcohol consumption, but do not reflect individual responses towards the intervention. Inter-individual variation has already been well established even for consumption of only the vehicle used in this study [3], and therefore, this aspect is not discussed in detail here. Instead, focus was placed on the individual, as well as the group, concentration changes of the five important metabolites that were significantly up-regulated during the whole time period following alcohol consumption, as well as on the concentration changes of uric acid, owing to its unique excretion profile.

**Fig 4:**
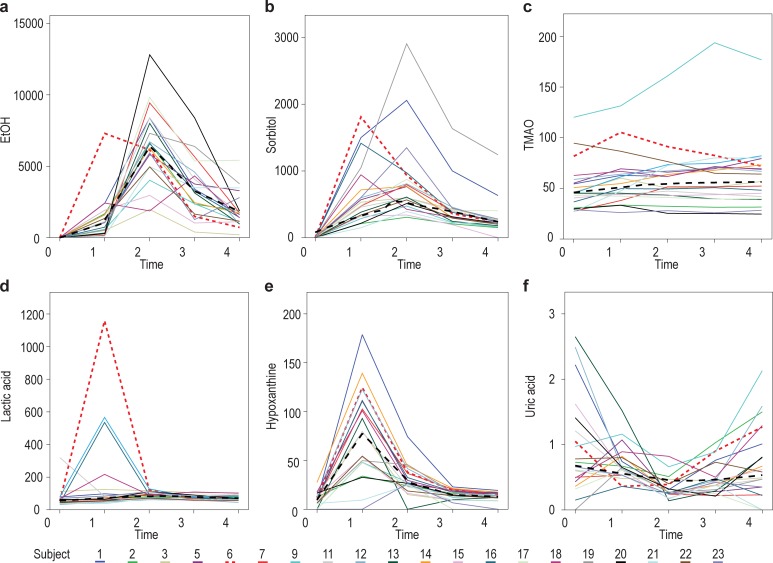
Changes in the concentrations of the six up-regulated metabolites from time 0 to time 4 following alcohol consumption. Each subplot displays a set of lines (coloured solid lines for 19 of the individuals and a red dotted line for one highlighted individual) representing the observations from the 20 individuals across 5 time points for a given metabolite: (a) ethanol; (b) sorbitol; (c) TMAO; (d) lactic acid; (e) hypoxanthine; and (f) uric acid. The black dashed lines represent one potential LOWESS regression for each metabolite against time.

Fig 4 displays subplots as a set of solid lines (and one dotted line) representing the observations from the 20 individuals across 5 time points for each of the six metabolites shown to be significantly up-regulated in Table 2. The black dashed lines represent one potential LOWESS (locally weighted scatterplot smoothing) regression for each metabolite against time using a bi-weight kernel. Most individuals excreted a limited amount of ethanol in their urine one hour after alcohol consumption, and, generally, ethanol excretion peaked two hours following alcohol consumption (Fig 4A). Likewise, most individuals showed a distinct excretion of the osmolyte sorbitol one hour after alcohol consumption (Fig 4B), albeit at different concentrations. The LOWESS regression curve for sorbitol, however, peaked at 2 hours for the group as a whole. The concentration of TMAO, another osmolyte, increased significantly from time 0 (Fig 4C), reaching a plateau at 3 hours. Its final concentration was, however, still higher (p = 0.037) than the baseline value at time 0.

The next observations from Fig 4 are closely associated with the known alcohol-induced disturbance of the NAD^+^:NADH ratio in hepatocytes. The excretion of lactic acid for the group peaked at 2 hours, but 4 individuals showed an early and excessive response one hour after alcohol consumption (Fig 4D). The profile of the LOWESS regression curve for hypoxanthine (Fig 4E) distinctly peaked one hour following alcohol consumption. Two of the early high lactic acid responders showed similar, high hypoxanthine excretion profiles. The general profile of uric acid excretion (Fig 4F) was a mirror image of that of hypoxanthine, indicating the NAD^+^-dependence of its formation through dehydrogenation of hypoxanthine (catalysed by xanthine dehydrogenase; EC 1.17.1.4).

Most individuals followed the general trend indicated by the LOWESS regression line, although one showed an early excretion of ethanol following alcohol consumption (shown as a red dotted line in Fig 4). Compared to the group, this individual also presented with a high excretion of sorbitol and TMAO, peaking at one hour, an early and extremely high lactic acid excretion, high excretion of hypoxanthine, but comparable excretion of uric acid. Since this person did not present as an outlier, we attribute this variation to the unique response of this individual to alcohol consumption. Diversity in the excretion profiles of the individuals within the group, emphasized by the one highlighted person, clearly illustrates the importance of inter-individuality, previously described for the consumption of only the vehicle [3]. Taken together, the combination of observations summarized in Table 2 and illustrated in Fig 4 indicate that the cases themselves are a noteworthy source of variation, while the group response provided the opportunity for the development of a holistic model of the effect of acute alcohol consumption.

**Fig 5:**
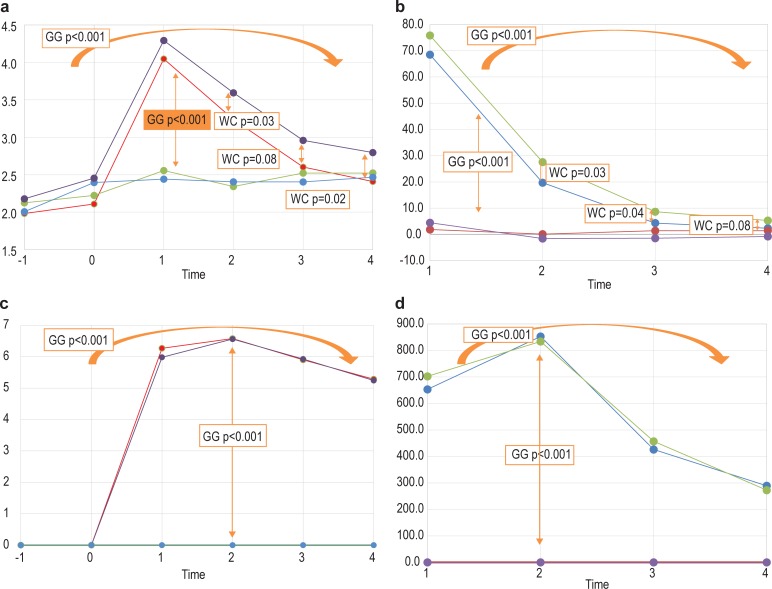
Indications of differences in the average levels of hypoxanthine and sorbitol across the four interventions and six time points. (a) Differences in the average levels of hypoxanthine from time –1 to time 4 between the four interventions. (b) Differences in the average change in hypoxanthine levels, measured from time 0, following the four interventions. (c and d) The comparative results for sorbitol. Significant differences were based on the Greenhouse–Geisser-corrected p-values from the RM ANOVA model or the Wilcoxon signed-rank tests, assessing differences between the sets of means, and are indicated by the arrows.

Finally, the ingestion of one tablet of an NAD-containing supplement, proposed to potentially counteract the effects of alcohol consumption, did not show a notable effect if taken one hour before the alcohol dose. To illustrate this, the complete design of this study (that is, four interventions measured over 6 time points) was modelled for quantified hypoxanthine and sorbitol, using a 2-way RM ANOVA model based on log transformed data to improve normality. Reported in the graphs are the Greenhouse–Geisser-corrected p-values for the main effects (where significant), as well as specific comparisons based on the Wilcoxon signed-rank tests (Section A.3.3 in S1 File provides more details on this analysis). These two metabolites were selected based on the NAD^+^-dependence of their catabolism (hypoxanthine → xanthine → uric acid, both reactions catalysed by xanthine dehydrogenase; and sorbitol → fructose, catalysed by sorbitol dehydrogenase (SD; EC 1.1.1.14), respectively). Fig 5 provides a brief overview of the information extracted from this model.

Fig 5A and 5C indicate a significant difference in the average levels of hypoxanthine and sorbitol, respectively, between the interventions including and excluding alcohol, as well as in time between the interventions including alcohol. Fig 5B and 5D, likewise, indicate a significant difference in the average change in the levels of hypoxanthine and sorbitol across the time period between the four interventions. All the results from this approach therefore arrive at the same conclusion—the observed differences were mainly attributable to the consumption of alcohol. For sorbitol, the differences were only significant when comparing the two interventions involving alcohol with the two not involving alcohol. No significant differences were observed between the two interventions not involving alcohol, nor between the two interventions involving alcohol. For hypoxanthine, significant differences were observed between the “alcohol plus vehicle” and the “vehicle plus alcohol and NAD” interventions at 2 and 3 hours after consumption, with significance on the average levels at 2 and 4 hours, respectively. Although alcohol consumption caused increased hypoxanthine excretion, it appears that the addition of NAD resulted in even higher amounts of hypoxanthine being excreted. These results indicate that NAD, as used in this study, might potentially have an effect in the later phases following alcohol consumption, but, as a whole, do not justify its application as a supplement to counteract the effects of alcohol consumption.

## Discussion

**Fig 6:**
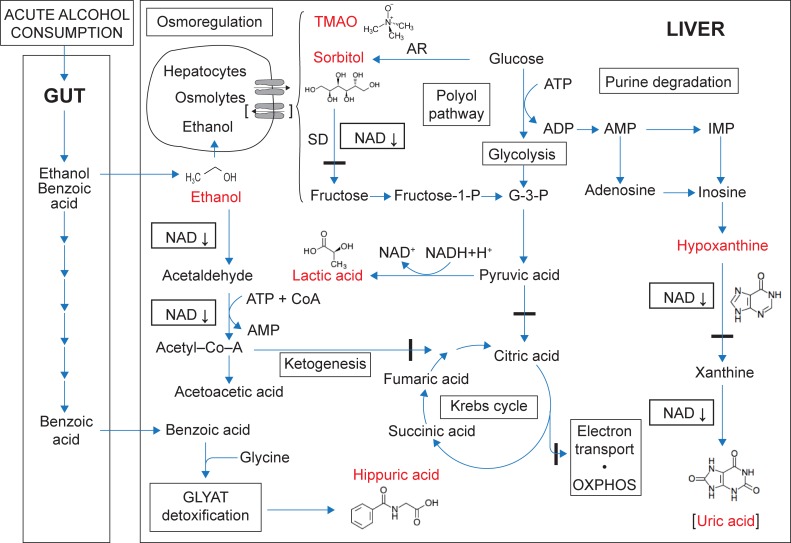
Model of the metabolite profile based on the important metabolites up-regulated following alcohol consumption. The main organs involved are the gut and the liver. Ethanol absorption is indicated in the upper region of the gut, and benzoic acid (from the vehicle and microbiome metabolism) in the lower gut. The main metabolism of ethanol, and the associated consequences of the disturbed NAD^+^:NADH ratio, occur in the liver. The structural formulas and names of the six important metabolites are shown in red (uric acid is indicated in brackets because it was not measured by NMR). Blue arrows are not related to enzyme kinetic reactions, but are used as indicators of the proposed flow directions following alcohol consumption. Osmoregulation is proposed as efflux in hepatocytes in the early phases following alcohol consumption, and potential influx (shown in brackets) in the later phases. Abbreviations: ATP, adenosine triphosphate; ADP, adenosine diphosphate; AMP, adenosine monophosphate; IMP, inosine monophosphate; G-3-P, glyceraldehyde-3-phosphate; GLYAT, glycine-N-acyltransferase; SD, sorbitol dehydrogenase; AR, aldose reductase; OXPHOS, oxidative phosphorylation.

It is known that three mechanisms for ethanol metabolism may be operative in the liver: (1) the ADH-pathway in the cytosol; (2) the microsomal ethanol oxidizing system (MEOS) of the endoplasmic reticulum; and (3) the catalase mechanism, located in peroxisomes [33]. In the ADH-pathway, ethanol is converted to acetaldehyde and acetate in two consecutive dehydrogenase reactions, both dependent on NAD^+^. During both of these reactions NAD^+^ is reduced, generating excess amounts of NADH. Following acute alcohol consumption, NAD^+^ depletion, and the subsequent excessive production of reducing equivalents, greatly perturb the hepatocyte redox homeostasis. This perturbation is known, but, through the present NMR metabolomics study, new insights were revealed—illustrated in Fig 6. This figure proposes a model that outlines three essential disturbances that occur following acute alcohol consumption—up-regulated lactic acid metabolism, down-regulated purine catabolism and osmoregulation.

First, up-regulation of lactic acid is coupled to the high hepatic NADH:NAD^+^ ratio (due to ADH- and ALDH-catalysed ethanol catabolism), which diverts pyruvic acid metabolism towards lactic acid, and subsequently inhibits gluconeogenesis. During low or chronic alcohol consumption, excess lactic acid is exported from the liver to peripheral tissues, where NADH levels are lower, and lactic acid may be reconverted to pyruvic acid for metabolic needs. During acute alcohol consumption, however, aerobic oxidation (that is, the Krebs cycle, the respiratory chain and oxidative phosphorylation) is known to be inhibited [5,6], which was indicated in our study by the increased levels of fumaric acid (not observed in all experimental subjects) and succinic acid. Similarly, due to the high hepatic NADH:NAD^+^ ratio and inhibition of the Krebs cycle, ethanol-derived acetyl-CoA may be converted to acetoacetic acid and 3-ketobutyric acid, as seen in the urine samples from some of our experimental subjects. The values of these metabolites were, however, generally too low to enable their quantification. A noteworthy observation was that a few of the experimental subjects showed excessive urinary lactic acid excretion shortly after alcohol intake (Fig 4D), whereas the excretion of the majority of the subjects peaked at two hours following alcohol consumption. These observations are yet a further example of the individual differences in coping with the consumed alcohol. The common paradigm is that variation in response to alcohol consumption is genetically controlled, and is suspected to cause a predisposition towards the development of alcohol-induced liver disease and alcoholism [34]. Furthermore, we observed that the mean value of urinary lactic acid peaked one hour after alcohol consumption (increasing roughly 2.5 times from 70.90 mM to 179.9 mM (*p* = 0.001)) and declined to a near normal value three hours after consumption (75.90 mM). This is comparable to the decreased lactic acid levels measured in liver and serum samples from rats, decapitated three hours following treatment with a single intragastric dose of ethanol [18].

Second, hypoxanthine, an intermediate in purine catabolism and a precursor of uric acid, appeared to be an important indicator of acute alcohol consumption (Fig 4E). This is in agreement with the increased hypoxanthine observed in patients suffering from alcohol- and hepatitis B-induced cirrhosis [11], and in those listed with stearamide as biomarker for hepatic cirrhosis [17]. In addition, several intravenous ethanol infusion, and related, studies on purine metabolism in humans (reviewed in [35]) indicated that many factors affect this metabolic pathway—daily drinking habits; the type of alcoholic beverages; exercise; and, ultimately, ALDH polymorphisms, are all important contributing factors. Increased AMP following alcohol consumption seems to be the important metabolic departure point in reflecting on purine catabolism—alcohol-induced diminished ATP production via glycolysis [36] and increased adenine nucleotide turnover [37], contribute towards increased ADP levels, and its increased conversion to AMP. Additionally, during the ADH- and ALDH-catalysed degradation of ethanol, two equivalents of ATP are consumed and two equivalents of AMP are produced for each equivalent of ethanol converted to acetyl-CoA [38]. All these perturbations lead to a considerable alcohol-induced increase in AMP, which is then catabolised and reflected in the hypoxanthine and, ultimately, uric acid profiles. Hyperuricaemia, gout and increased urinary uric acid excretion, accompanied by raised urinary hypoxanthine and xanthine concentrations, are accepted indicators of chronic alcohol consumption, although excretion of hypoxanthine was shown to be lower than that of xanthine in regular drinkers [39]. In our study, we observed a highly significant increase (Table 2: p ≤ 0.0001; FC = +8.187) in hypoxanthine excretion in all 20 cases studies, which peaked at one hour after alcohol consumption in 18 of the cases, and at 2 hours in the other two cases (Fig 4E). The general pattern of the urinary excretion of uric acid (Fig 4F) presents as a mirror image to that of hypoxanthine, initially decreasing and then returning to near its starting concentration. We relate these observations to the alcohol-induced perturbed hepatic redox state—depleted NAD^+^, the cofactor for xanthine dehydrogenase, interrupts the purine catabolic pathway, decreasing the conversions of hypoxanthine to xanthine, and xanthine to uric acid. However, as the NADH:NAD^+^ ratio normalizes, these reactions can once again take place, resulting in the catabolism of hypoxanthine to produce uric acid. It should, however, be noted that lactic acid, which is highly increased following alcohol consumption, competitively inhibits the clearance of uric acid through the renal proximal tubule [40], which further supports the observed initial decrease in uric acid excretion following alcohol consumption. Thus, while hyperuricaemia is an accepted marker of regular and chronic alcohol consumption, increased excretion of hypoxanthine may possibly act as an indicator of acute alcohol consumption.

Third, of notable interest, was the observation of the increased urinary excretion of sorbitol following ethanol consumption, which is not listed in the review on global metabolic profiling studies on alcohol-related disorders, covering NMR, GC–MS and LC–MS approaches [17]. Sorbitol was not detected in any of the urine samples collected prior to any of the interventions (normal reference value: 3.506 ± 2.24 μmol/mmol creatinine) [25]. Its excretion, however, increased rapidly in most individuals following alcohol consumption (Fig 4B). Moreover, the sorbitol excretion profile did not seem to be affected by the consumption of the NAD supplement, taken prior to alcohol consumption (Fig 5C and 5D). Sorbitol is an organic osmolyte present in all human cells, and, together with other osmolytes, reaches very high concentrations (in the millimolar range) in the cytosol. In human cells, the osmolytes are classified into three groups [41]: (1) amino acids and their derivatives (including taurine); (2) methylamines (including TMAO); and (3) polyols (including sorbitol). Osmolytes play key roles as cytoprotectants, and in maintaining cell volume homeostasis [42]. They function as nonperturbing solutes, which permits their accumulation to high levels and large shifts in their concentrations without having deleterious effects on cellular structure and function [41]. These unique characteristics of osmolytes opened several lines of thought regarding the perturbation of sorbitol following acute alcohol consumption. Of these postulates, sorbitol synthesis and catabolism (occurring in the polyol pathway), as well as its function in osmoregulation, are pertinent:

(1) The NADPH-dependent enzyme aldose reductase (AR; EC 1.1.1.21) catalyses the synthesis of sorbitol from glucose. This reaction is highly operative under hyperglycaemic conditions, such as in diabetes mellitus [43], when up to 30% of glucose is channelled into the AR-catalysed polyol pathway. However, given the fasting state of the experimental subjects before the “alcohol plus vehicle” intervention, and the increased anaerobic oxidation of glucose towards lactic acid, it seems unlikely that sorbitol accumulation was due to the activation of sorbitol synthesis. (2) The next step in the polyol pathway is the degradation of sorbitol to fructose, a reaction catalysed by the NAD^+^-dependent enzyme sorbitol dehydrogenase (SD). In this study, sorbitol accumulation may be attributed to the alcohol-induced inhibition of its catabolism—due to the metabolism of ethanol, the NAD^+^ required for the dehydrogenation of sorbitol becomes depleted. Furthermore, significant down-regulation of SD has been observed in a study on the changes of the cytoplasmic proteome in response to alcoholic hepatotoxicity in rats [44], which lends support to this viewpoint. (3) Increased sorbitol as a consequence of its role in osmoregulation, however, seems to be the preferred explanation for interpreting the observed urinary excretion profile of sorbitol. Ethanol is both water and lipid soluble, which renders it a membrane-permeable substance. Abnormal cell volume regulation significantly contributes to the pathophysiology of several disorders, and cells respond to these changes by importing, exporting, or synthesizing osmolytes to maintain volume homeostasis [45]. On an experimental level, oedema/cell swelling could be induced by binge-simulated ethanol exposure in slice cultures of the developing rat brain [46]. In a cell-to-medium flux study, hyperosmotically induced intracellular accumulation of sorbitol in renal epithelial cells showed a greater than 150-fold increased efflux within five minutes after exposure to an isosmotic medium. We thus speculate that the increased sorbitol excretion in our alcohol-exposed subjects relates to ethanol-induced hepatocyte swelling, which is compensated for by sorbitol release in order to maintain cellular volume homeostasis. Although less pronounced in this study than that of sorbitol, perturbations of other osmolytes, such as TMAO and taurine (observed in this study, see Table 2), should also be considered in the examination of osmolyte responses.

In summary, we have described the urinary metabolite profile of healthy, young males following acute alcohol consumption as part of a designed intervention study. The complex NMR spectral data, generated from individuals participating in a time-dependent cross-over study, could be resolved sufficiently through the application of univariate and multivariate statistical analyses. This approach provided a novel method for the analysis and understanding of the complex metabolomics data that were produced due to acute alcohol consumption. We indicate that NAD^+^ depletion, and the production of an excessive amount of reducing equivalents, greatly perturb the hepatocyte redox homeostasis, resulting in metabolic disturbances, of which urinary excretion of sorbitol is novel. We postulate that sorbitol is a marker of a cell volume regulatory response to ethanol-induced hepatocyte swelling. This may have a wider significance related to brain oedema-induced neurodegenerative damage following chronic binge alcohol exposure [46,47].

## Supporting information

S1 File Details of sample preparation, ^1^H-NMR analysis and data analysis methods. **(**Section A) Method for sample treatment, storage, preparation and ^1^H-NMR analysis. **(**Section A.1) Sample collection, characterization and storage. **(**Section A.2) Sample preparation and ^1^H-NMR analysis. **(**Section A.3) Data processing. **(**Section A.3.1) Pre-processing. **(**Section A.3.2) Quality assurance. **(**Section A.3.3) The interaction effect of NAD and alcohol. (Section B) Uric acid analysis. **(**Section C) Original ^1^H-NMR spectral data References.
(PDF)

S2 File Raw ^1^H-NMR spectral data are given as an electronic file in excel format. (XLSX)

## References

[1] Gibney MJ, Walsh M, Brennan L, Roche HM, German B, Van Ommen B. Metabolomics in human nutrition: opportunities and challenges. Am J Clin Nutr. 2005; 82:497–503. doi: 10.1093/ajcn.82.3.497 16155259 10.1093/ajcn.82.3.497

[2] Wishart DS. Metabolomics: applications to food science and nutrition research. Trends Food Sci Tech. 2008; 19:482–493.

[3] Irwin C, van Reenen M, Mason S, Mienie LJ, Westerhuis JA, Reinecke CJ. Contribution towards a metabolite profile of the detoxification of benzoic acid through glycine conjugation: an intervention study. PLOS ONE. 2016; 11(12):e0167309 doi: 10.1371/journal.pone.0167309 27907139 10.1371/journal.pone.0167309 PMC5132330

[4] Bradford BU, Rusyn I. Swift increase in alcohol metabolism (SIAM): understanding the phenomenon of hypermetabolism in liver. Alcohol. 2005; 35(1):13–17. doi: 10.1016/j.alcohol.2004.12.001 15922133 10.1016/j.alcohol.2004.12.001

[5] Hawkins RD, Kalant H.The metabolism of ethanol and its metabolic effects. Pharmacol Rev. 1972; 24(1):67–157. 4402043

[6] Zakhari S, Li T-K. Determinants of alcohol use and abuse: impact of quantity and frequency patterns on liver disease. Hepatology. 2007; 46(6):2032–2039. doi: 10.1002/hep.22010 18046720 10.1002/hep.22010

[7] Lieber CS. Ethanol metabolism, cirrhosis and alcoholism. Clin chim acta. 1997; 257(1):59–84. 9028626 10.1016/s0009-8981(96)06434-0

[8] Nahon P, Amathieu R, Triba MN, Bouchemal N, Nault JC, Ziol M, et al Identification of serum proton NMR metabolomic fingerprints associated with hepatocellular carcinoma in patients with alcoholic cirrhosis. Clin Cancer Res. 2012; 18(24):6714–6722. doi: 10.1158/1078-0432.CCR-12-1099 23136190 10.1158/1078-0432.CCR-12-1099

[9] Rachakonda V, Gabbert C, Raina A, Bell LN, Cooper S, Malik S, et al Serum metabolomic profiling in acute alcoholic hepatitis identifies multiple dysregulated pathways. PLOS ONE. 2014; 9(12):e113860 doi: 10.1371/journal.pone.0113860 25461442 10.1371/journal.pone.0113860 PMC4252257

[10] Amathieu R, Triba MN, Nahon P, Bouchemal N, Kamoun W, Haouache H, et al Serum 1H-NMR metabolomic fingerprints of acute-on-chronic liver failure in intensive care unit patients with alcoholic cirrhosis. PLOS ONE. 2014; 9(2):e89230 doi: 10.1371/journal.pone.0089230 24586615 10.1371/journal.pone.0089230 PMC3929651

[11] Lian JS, Liu W, Hao SR, Guo YZ, Huang HJ, Chen DY, et al A serum metabonomic study on the difference between alcohol- and HBV-induced liver cirrhosis by ultraperformance liquid chromatography coupled to mass spectrometry plus quadrupole time-of-flight mass spectrometry. Chin Med J. 2011; 124(9):1367–1373. 21740750

[12] Gika HG, Ji C, Theodoridis GA, Michopoulos F, Kaplowitz N, Wilson ID. Investigation of chronic alcohol consumption in rodents via ultra-high-performance liquid chromatography–mass spectrometry based metabolite profiling. J Chromatogr A. 2012; 1259:128–137. doi: 10.1016/j.chroma.2012.02.053 22446076 10.1016/j.chroma.2012.02.053 PMC3387526

[13] Loftus N, Barnes A, Ashton S, Michopoulos F, Theodoridis G, Wilson I, et al Metabonomic investigation of liver profiles of nonpolar metabolites obtained from alcohol-dosed rats and mice using high mass accuracy MSn analysis. J Proteome Res. 2010; 10(2):705–713. doi: 10.1021/pr100885w 21028815 10.1021/pr100885w PMC3033970

[14] Gao X, Zhao A, Zhou M, Lin J, Qiu Y, Su M, et al GC/MS-based urinary metabolomics reveals systematic differences in metabolism and ethanol response between Sprague–Dawley and Wistar rats. Metabolomics. 2011; 7(3) 363–374.

[15] Kirpich IA, Petrosino J, Ajami N, Feng W, Wang Y, Liu Y, et al Saturated and unsaturated dietary fats differentially modulate ethanol-induced changes in gut microbiome and metabolome in a mouse model of alcoholic liver disease. Am J Pathol. 2016; 186:765–776. doi: 10.1016/j.ajpath.2015.11.017 27012191 10.1016/j.ajpath.2015.11.017 PMC5808146

[16] Manna SK, Patterson AD, Yang Q, Krausz KW, Idle JR, Fornace Jr. AJ, et al UPLC–MS-based urine metabolomics reveals indole-3-lactic acid and phenyllactic acid as conserved biomarkers for alcohol-induced liver disease in the *Ppara*-null mouse model. J Proteome Res. 2011; 10(9), 4120–4133. doi: 10.1021/pr200310s 21749142 10.1021/pr200310s PMC3170755

[17] Gika HG, Wilson ID. Metabolic profiling approaches for biomarkers of ethanol intake In: Patel VB, editor. Molecular aspects of alcohol and nutrition. Academic Press; 2016 pp. 213–222.

[18] Nicholas PC, Kim D, Crews FT, Macdonald JM. 1H NMR-based metabolomic analysis of liver, serum, and brain following ethanol administration in rats. Chem Res Toxicol. 2008; 21:408–420. doi: 10.1021/tx700324t 18095657 10.1021/tx700324t

[19] Bradford BU, O'Connell TM, Han J, Kosyk O, Shymonyak S, Ross PK, et al Metabolomic profiling of a modified alcohol liquid diet model for liver injury in the mouse uncovers new markers of disease. Toxicol Appl Pharmacol. 2008; 232:236–243. doi: 10.1016/j.taap.2008.06.022 18674555 10.1016/j.taap.2008.06.022 PMC2583460

[20] Masuo Y, Imai T, Shibato J, Hirano M, Jones OAH, Maguire ML, et al Omic analyses unravels global molecular changes in the brain and liver of a rat model for chronic Sake (Japanese alcoholic beverage) intake. Electrophoresis. 2009; 30:1259–1275. doi: 10.1002/elps.200900045 19382137 10.1002/elps.200900045

[21] Fernando H, Kondraganti S, Bhopale KK, Volk DE, Neerathilingam M, Kaphalia BS, et al 1H and 31P NMR lipidome of ethanol-induced fatty liver. Alcohol Clin Exp Res. 2010; 34(11):1937–1947. doi: 10.1111/j.1530-0277.2010.01283.x 20682011 10.1111/j.1530-0277.2010.01283.x PMC3095964

[22] Yoseph BP, Breed E, Overgaard CE, Ward CJ, Liang Z, Wagener ME, et al Chronic alcohol ingestion increases mortality and organ injury in a murine model of septic peritonitis. PLOS ONE. 2013; 8(5):e62792 doi: 10.1371/journal.pone.0062792 23717394 10.1371/journal.pone.0062792 PMC3661585

[23] Vázquez-Fresno R, Llorach R, Alcaro F, Rodríguez MA, Vinaixa M, Chiva-Blanch G, et al (1)H-NMR-based metabolomic analysis of the effect of moderate wine consumption on subjects with cardiovascular risk factors. Electrophoresis. 2012; 33(15):2345–2354. doi: 10.1002/elps.201100646 22887155 10.1002/elps.201100646

[24] Würtz P, Cook S, Wang Q, Tiainen M, Tynkkynen T, Kangas AJ, et al Metabolic profiling of alcohol consumption in 9778 young adults. Int J Epidemiol. 2016; 45(5):1493–1506. doi: 10.1093/ije/dyw175 27494945 10.1093/ije/dyw175 PMC5100616

[25] Bouatra S, Aziat F, Mandal R, Guo AC, Wilson MR, Knox C, et al The human urine metabolome. PLOS ONE. 2013; 8(9):e73076 doi: 10.1371/journal.pone.0073076 24023812 10.1371/journal.pone.0073076 PMC3762851

[26] Bala S, Marcos M, Gattu A, Catalano D, Szabo G. Acute binge drinking increases serum endotoxin and bacterial DNA levels in healthy individuals. PLOS ONE. 2014; 9(5): e96864 doi: 10.1371/journal.pone.0096864 24828436 10.1371/journal.pone.0096864 PMC4020790

[27] Antti H, Bollard ME, Ebbels T, Keun H, Lindon JC, Nicholson JK, et al Batch statistical processing of 1NMR-derived urinary spectral data. J Chemometrics. 2002; 16:461–468.

[28] Smilde AK, Jansen JJ, Hoefsloot HCJ, Lamers R-JAN, Van der Greef J, Timmerman ME. ANOVA-simultaneous component analysis (ASCA): a new tool for analyzing designed metabolomics data. Bioinformatics. 2005; 21:3043–3048. doi: 10.1093/bioinformatics/bti476 15890747 10.1093/bioinformatics/bti476

[29] World Health Organization. Global status report on alcohol and health 2014. http://www.who.int/substance_abuse/publications/global.alcohol_report/en/ Date of access: 24 Aug. 2017.

[30] Numminen H, Syrjälä M, Benthin G, Kaste M, Hillborn M. The effect of acute ingestion of a large dose of alcohol on the hemostatic system and its circadian variation. Stroke. 2000, 31(6):1269–1273 10835443 10.1161/01.str.31.6.1269

[31] Viant M, Bearden DW, Bundy JG, Burton IW, Colette TW, Ekman DR, et al International NMR-based environmental metabolomics intercomparison exercise. Environ Sci Technol. 2009; 43:219–225. 19209610 10.1021/es802198z

[32] Westerhuis JA, van Velzen EJJ, Hoefsloot HCJ, Smilde AK. Multivariate paired data analysis: multilevel PLSDA versus OPLSDA. Metabolomics. 2010; 6(1):119–128 doi: 10.1007/s11306-009-0185-z 20339442 10.1007/s11306-009-0185-z PMC2834771

[33] Lieber CS. Alcohol and the liver: 1994 update. Gastroenterology. 1994; 106(4):1085–1105. 8143977 10.1016/0016-5085(94)90772-2

[34] Day CP, Bashir R, James OF, Bassendine MF, Crabb DW, Thomasson HR, et al Investigation of the role of polymorphisms at the alcohol and aldehyde dehydrogenase loci in genetic predisposition to alcohol‐related end‐organ damage. Hepatology. 1991; 14(5):798–801. 1937384 10.1002/hep.1840140509

[35] Yamamoto T, Moriwaki Y, Takahashi S. Effect of ethanol on metabolism of purine bases (hypoxanthine, xanthine, and uric acid). Clin Chim Acta. 2005; 356(1):35–57. 15936302 10.1016/j.cccn.2005.01.024

[36] Yuki T, Thurman RG. The swift increase in alcohol metabolism: Time course for the increase in hepatic oxygen uptake and the involvement of glycolysis. Biochem J. 1980; 186(1):119–126. 6989357 10.1042/bj1860119 PMC1161510

[37] Faller J, Fox IH. Ethanol-induced hyperuricemia: evidence for increased urate production by activation of adenine nucleotide turnover. N Engl J Med. 1982; 307(26):1598–1602. doi: 10.1056/NEJM198212233072602 7144847 10.1056/NEJM198212233072602

[38] Puig JG, Fox IH. Ethanol-induced activation of adenine nucleotide turnover: Evidence for the role of acetate. J Clin Invest. 1984; 74(3):936–941. doi: 10.1172/JCI111512 6470146 10.1172/JCI111512 PMC425250

[39] Nishimura T, Shimizu T, Mineo I, Kawachi M, Ono A, Nakajima H, et al Influence of daily drinking habits on ethanol-induced hyperuricemia. Metabolism. 1994; 43(6):745–748. 8201965 10.1016/0026-0495(94)90125-2

[40] Yü TF, Sirota JH, Berger L, Halpern M, Gutman AB. Effect of Sodium Lactate Infusion on Urate Clearance in Man. Proc Soc Exp Biol Med. 1957; 96(3):809–813. 13505867 10.3181/00379727-96-23616

[41] Strange K. Cellular volume homeostasis. Adv Physiol Educ. 2004; 28:155–159. doi: 10.1152/advan.00034.2004 15545344 10.1152/advan.00034.2004

[42] Lang F, Busch GL, Ritter M, Völkl H, Waldegger S, Gulbins E, et al Functional significance of cell volume regulatory mechanisms. Physiol rev. 1998; 78(1):247–306. doi: 10.1152/physrev.1998.78.1.247 9457175 10.1152/physrev.1998.78.1.247

[43] Tang WH, Martin KA, Hwa J. Aldose reductase, oxidative stress, and diabetic mellitus. Front Pharmacol. 2012; 3:87 doi: 10.3389/fphar.2012.00087 22582044 10.3389/fphar.2012.00087 PMC3348620

[44] Kim DH, Lee E-M, Do S-H, Jeong D-H, Jeong KS. Changes of the Cytoplasmic Proteome in Response to Alcoholic Hepatotoxicity in Rats. Int J Mol Sci. 2015; 16:18664–18682. doi: 10.3390/ijms160818664 26266409 10.3390/ijms160818664 PMC4581265

[45] Diamond I, Gordon AS. Cellular and molecular neuroscience of alcoholism. Physiol rev. 1997; 77(1):1–20. doi: 10.1152/physrev.1997.77.1.1 9016298 10.1152/physrev.1997.77.1.1

[46] Moon KH, Tajuddin N, Brown J, Neafsey EJ, Kim HY, Collins MA. Phospholipase A2, Oxidative Stress, and Neurodegeneration in Binge Ethanol‐Treated Organotypic Slice Cultures of Developing Rat Brain. Alcohol Clin Exp Res. 2014; 38(1):161–169. doi: 10.1111/acer.12221 23909864 10.1111/acer.12221 PMC3866207

[47] Collins MA, Zou JY, Neafsey EJ. Brain damage due to episodic alcohol exposure in vivo and in vitro: furosemide neuroprotection implicates edema-based mechanism. FASEB J. 1998; 12:221–230. 9472987 10.1096/fasebj.12.2.221

