# Supplementary material for: The 1H-NMR-based metabolite profile of acute alcohol consumption: A metabolomics intervention study
Source: PLoS One. 2018 May 10;13(5):e0196850. doi: 10.1371/journal.pone.0196850 (PMC5944960; doi:10.1371/journal.pone.0196850)
Supplement: S1 File — (Section A) Method for sample treatment, storage, preparation and 1H-NMR analysis. (Section A.1) Sample collection, characterization and storage. (Section A.2) Sample preparation and 1H-NMR analysis. (Section A.3) Data processing. (Section A.3.1) Pre-processing. (Section A.3.2) Quality assurance. (Section A.3.3) The interaction effect of NAD and alcohol. (Section B) Uric acid analysis. (Section C) Original 1H-NMR spectral data References. (PDF) [file pone.0196850.s001.pdf]

# The $^1$H-NMR-based metabolite profile of acute alcohol consumption: a metabolomics intervention study — Supporting Information

Cindy Irwin[1¶], Mari van Reenen[1,2¶], Shayne Mason[1], Lodewyk J. Mienie[1], Ron A. Wevers[3], Johan A. Westerhuis[2,4] and Carolus J. Reinecke[1*]

[1]Centre for Human Metabolomics, Faculty of Natural Sciences and Agriculture, North-West University (Potchefstroom Campus), Potchefstroom, South Africa

[2]Department of Statistics, Faculty of Natural Sciences and Agriculture, North-West University (Potchefstroom Campus), Potchefstroom, South Africa

[3]Department of Laboratory Medicine, Radboud University Nijmegen Medical Centre, Nijmegen, The Netherlands

[4]Biosystems Data Analysis, Swammerdam Institute for Life Sciences, University of Amsterdam, Amsterdam, The Netherlands

*Corresponding author
E-mail: carools.reinecke@nwu.ac.za (CJ)

[¶]These authors contributed equally to this work

# Section A Methods for sample treatment, storage, preparation and $^{1}$H-NMR analysis

## Section A.1 Sample collection, characterization and storage

All the subjects were randomly assigned to one of the four intervention groups until all 24 had participated in all four interventions. Six urine samples were collected from each subject for each intervention, giving a total of 24 samples from each subject over the course of the study.

One 5 mL and two 1 mL vials were used to provide aliquots of each of the urine samples; these aliquots, together with the remainder of the bulk urine samples, were stored at –80ºC. Once all the urine samples were collected, one 1 mL aliquot of each was thawed and combined to prepare a pooled quality control (QC) sample for the experiment as a whole. This QC sample was then divided into 15 mL aliquots and once again stored at –80ºC.

The collected samples were analysed in 24 separate batches, each batch containing the 24 samples of a single subject and three QC samples. The 27 samples from each of the 24 batches were analysed in the following order:

$QC_1\ [S_{-1}S_0S_1S_2S_3S_4]_{Vehicle}\ [S_{-1}S_0S_1S_2S_3S_4]_{Alcohol}\ QC_2\ [S_{-1}S_0S_1S_2S_3S_4]_{NAD}\ [S_{-1}S_0S_1S_2S_3S_4]_{NAD+Alcohol}\ QC_3$

where $S_{-1}$ represents the sample collected at time –1, $S_0$ represents the sample collected at time 0, and so on.

## Section A.2 Sample preparation and $^1$H-NMR analysis

Spectral analyses were conducted at the NMR facility of the Centre for Human Metabolomics at North-West University. Prior to analysis, an aqueous 1.5 M $KH_2PO_4$ deuterated buffer solution at pH 7.4 was prepared [1]. This solution served to lock the signal during analysis, maintained a stable pH in the sample and contained trimethyl-2,2,3,3-tetradeuteropropionic acid (TSP, sodium salt; Sigma Aldrich) as the internal standard to provide a chemical shift reference of δ = 0.00. The urine samples, stored at –80$^o$C, were thawed at room temperature for analysis. A 600 µL volume of each sample was centrifuged at 12 000 × g for 5 min to remove any sediments or debris. A 60 µL volume of buffer solution was added to 540 µL of the supernatant, vortexed and transferred to a 5-mm NMR tube.

Each sample so prepared was analysed on a Bruker Avance III HD 500 MHz NMR spectrometer equipped with a triple-resonance inverse (TXI) $^1$H{$^{15}$N,$^{13}$C} probe head and x, y, z gradient coils. $^1$H spectra were acquired as 128 transients in 32K data points with a spectral width of 6002 Hz. The sample temperature was maintained at 300 K and the $H_2O$ resonance was pre-saturated by single-frequency irradiation during a relaxation delay of 4 s, with a 90° excitation pulse fixed at 8 µs. Shimming of the sample was performed automatically on the deuterium signal. The resonance line widths for TSP and metabolites were <1 Hz (measurements at half the height of the peak). Fourier transformation and phase and baseline correction were done automatically. The software used was Bruker Topspin (V3.2) and Bruker AMIX (V3.9.9) [2].

All urine samples were normalized with reference to the creatinine $CH_2$ peak at 4.05 ppm. We employed two methods of spectral analysis: (1) Applying

equidistant binning (using a bin width of 0.02 ppm) to the spectral region between 0.5 and 10 ppm, excluding the region of the water peak (4.72–4.88 ppm). This gave a total of 467 integrated units per NMR spectrum for each sample for statistical analysis — spectral data. (2) Using information from the statistical outputs to identify discriminatory metabolites (based upon pure compound spectral libraries), and then accurately integrating the selected metabolites — providing quantified concentration data (μmol metabolite/mmol creatinine) for univariate analysis (see Table 2 in the main text).

# Section A.3 Data processing

## A.3.1 Pre-processing

The use of NMR spectroscopy as a tool for metabolomics is limited by the sensitivity (limit of detection) of NMR [3], requiring an approach in discarding noise while retaining and then measuring real peaks. The spectral intensities within the original 467 bins generated here ranged from $7.8 \times 10^5$ to $1.7 \times 10^9$. Based on previous empirical experience with NMR spectral analysis, we defined a threshold value of $2 \times 10^6$, being approximately the limit of detection of metabolomic substances presumed to be present in a spectral bin. To reduce the uncertainty in the data at the level of the approximate detection limit, we applied the threshold of $2 \times 10^6$ to the data matrix, setting all values below this threshold to zero. We subsequently applied a “zero-filter” to remove bins with more than 50% zero observations across all six time points, as applied for fusion of MS-based metabolomics data [4]. This resulted in a reduction in the number of bins containing spectral data from 467 to 347.

The remaining zero observations were replaced by random numbers below the minimum non-zero observation for each bin, after which the data were log scaled and auto scaled.

To account for the dilution differences between the urine samples, the value of each bin was normalized relative to the $CH_2$ peak of creatinine. This peak is contained in the 4.05 and 4.07 ppm bins. Therefore, each bin value was divided by the sum of the value in these two bins to normalize the bin values relative to creatinine. After normalization, the three bins related to creatinine (4.05, 4.07 and 3.05 ppm) were also removed from the matrix, giving a final total of 344 bins containing spectral data.

### A.3.2 Quality assurance

Quality control (QC) samples were included in each batch at the start, middle and end if the analytical run.  The variation and correlation within and between bins for QC and experimental samples were compared using principal component analysis (PCA) to attain a birds-eye view of any batch effects. NMR data are not known typically to present with such variation structures (that is, within or between batch effects), and this was also the case here. The PCA scores plot (Figure A) shows a similar correlation structure for the QC and experimental samples, but much less variation in the QC samples. From these results it was assumed that no batch corrections were required.

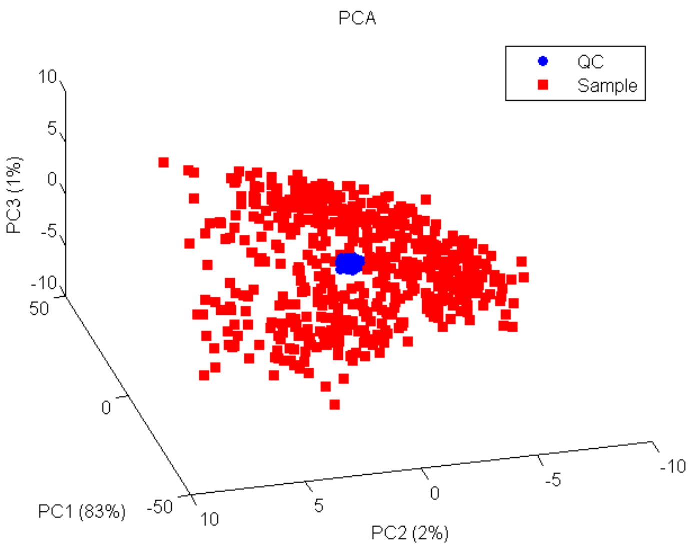


**Figure A. PCA scores plot illustrating the variation and correlation within and between bins for QC and experimental samples.** The close clustering of the QC samples compared to the experimental samples indicates that the variation in the QC samples is much less than the variation in the experimental samples. These results suggest that no batch corrections were required.

### A.3.3 The interaction effect of NAD and alcohol

As explained in the main text, the complete design of the study (that is, four intervention measured over 6 time points across all individuals) was modelled for quantified hypoxanthine and sorbitol using a two-way repeated measures analysis of variance (2-way RM ANOVA) model. A brief overview of the information extracted from the model is presented in Fig 5 of the main text. The absence of these metabolites prior to the intervention and sudden increase in their levels shortly thereafter, resulted in a high frequency of zero observations, thus introducing a spike in the distribution of the data. Therefore, the nature of the experiment and metabolites under investigation resulted in the data not being normally distributed. To account for this, log transformed data were used to build the RM ANOVA model, while Greenhouse–Geisser-corrected p-values were used to assess the significance of the main effects (that is, intervention and time). Unfortunately, inspection of the

residuals indicated that the transformation was not able to produce normality across all effect levels. The Wilcoxon signed-rank test was used as a confirmation test on the untransformed data to test for significantly different pairs of means as indicated in Figs 5a and 5c in the main text. However, since the performance of the Wilcoxon signed-rank test may also be compromised due to the spike in the data, difference data were assessed as a final confirmation to provide additional proof of the differences observed. The difference data were created by subtracting time 0 data from the remaining time points, while ignoring time –1. Doing so significantly improved the distribution of the data. The results based on the difference data are summarised in Figs 5b and 5d in the main text.

# Section B Uric acid analysis

The manual of the protocol detailing the uric acid analysis is available online as a downloadable PDF at https://www.thermofisher.com/order/catalog/product/ TR24321 [5].

## Infinity™
## Uric Acid Liquid Stable Reagent

### PRODUCT SUMMARY

| | | |
|---|---|---|
| Stability | : | Until Expiry at 2 - 8°C |
| Linear Range | : | 0.03-1.5 mmol/L (0.5-25.2 mg/dL) |
| Specimen Type | : | Serum or Urine |
| Method | : | Enzymatic Endpoint |
| Reagent Preparation | : | Supplied ready to use. |

IVD

### SYMBOLS IN PRODUCT LABELLING

| | | | |
|---|---|---|---|
| EC REP | Authorized Representative | | Temperature Limitation |
| IVD | For in vitro diagnostic use | | Use by/Expiration Date |
| LOT | Batch code/Lot number | | CAUTION. CONSULT INSTRUCTIONS FOR USE. |
| REF | Catalogue number | | Manufactured by |
| i | Consult instructions for use | | |

**INTENDED USE**
This reagent is intended for the in vitro quantitative determination of Uric Acid in human serum or urine.

**CLINICAL SIGNIFICANCE**
Uric acid is a metabolite of purines, nucleic acids and nucleoproteins: consequently, abnormal levels may be indicative of a disorder in the metabolism of these substances. Hyperuricaemia may be observed in renal dysfunction, gout, leukemia, polycythaemia, atherosclerosis, diabetes, hypothyroidism, or in some genetic diseases. Decreased levels are present in patients with Wilson's Disease. [1, 2, 3]

**METHODOLOGY**
This reagent is based upon the methods of Trivedi and Kabasakalian[4,5] with a modified Trinder[6] peroxide assay using 2,4,6-Tribromo-3-hydroxy benzoic acid (TBHB).

The series of reactions involved in the assay system is as follows:

1. $\text{Uric Acid} + O_2 + H_2O \xrightarrow{\textit{Uricase}} \text{Allantoin} + CO_2 + H_2O_2$

2. $2H_2O_2 + \text{4-AAP} + \text{TBHB} \xrightarrow{\textit{Peroxidase}} \text{Quinoneimine} + H_2O$

1. Uric Acid is oxidised to allantoin by uricase with the production of $H_2O_2$.
2. The peroxide reacts with 4-aminoantipyrine (4-AAP) and TBHB in the presence of peroxidase to yield a quinoneimine dye. The resulting change in absorbance at 520nm (520-550nm) is proportional to uric acid concentration in the sample.

**REAGENT COMPOSITION**

| Active Ingredients | Concentration |
|---|---|
| 4-Aminoantipyrine | 0.5 mmol/L |
| TBHB | 1.75 mmol/L |
| Uricase (Bacillus Sp.) | > 120 U/L |
| Peroxidase (Horseradish) | > 500 U/L |
| Tris Buffer | 50 mmol/L |

pH 8.25 ± 0.1 at 20°C.

***WARNING:*** Do not ingest. Avoid contact with skin and eyes. If spilt thoroughly wash affected areas with water. Reagent contains Sodium Azide which may react with copper or lead plumbing. Flush with plenty of water when disposing. For further information consult the Infinity Uric Acid Liquid Stable Reagent Material Safety Data Sheet.

**CAUTION:** This product contains animal source material. Handle and dispose of this product as if it were potentially infectious.

**REAGENT PREPARATION**
Reagent is supplied ready to use.

**STABILITY AND STORAGE**
When stored refrigerated at 2-8°C the reagent is stable until the expiry date stated on the bottle and kit box label.

***Indications of Reagent Deterioration:***
- Turbidity;
- Reagent Absorbance >0.5 AU at 520nm; and/or
- Failure to recover control values within the assigned range.

**SPECIMEN COLLECTION AND HANDLING**
**Collection:** No special preparation of the patient is required.
**Serum:** Use non-haemolysed serum.
**Urine:** It is recommended that 15 mL of 2 mol/L NaOH be added to the collection vessel. Upon receipt of the urine sample, pH should be checked. If the pH is less than 8.0 it should be adjusted with NaOH. A 1:10 dilution of urine is typically required prior to analysis. [7]
**Storage:** Serum samples are stable for at least 3 days at room temperature (18-25°C) and for at least 6 months frozen.[2] Stabilized urine may be stored at room temperature for 5 days.[7]

**ADDITIONAL EQUIPMENT REQUIRED BUT NOT PROVIDED**
- If required, pipettes for accurately dispensing measured volumes.
- A clinical chemistry analyzer capable of maintaining constant temperature (37°C) and measuring absorbance at 520 nm.
- Analyzer specific consumables, eg: sample cups.
- Normal and abnormal assayed control material.
- Calibrator or a suitable aqueous standard (see calibration section).

**ASSAY PROCEDURE**
The following system parameters are recommended. Individual instrument applications are available upon request from the Technical Support Group.

**SYSTEM PARAMETERS**

| | | |
|---|---|---|
| Temperature | | 37°C |
| Primary Wavelength | | 520 nm (520-550 nm) |
| Secondary Wavelength | | 600 - 660 nm |
| Assay Type | | Endpoint |
| Direction | | Increase |
| Sample : | Reagent Ratio | 1 : 50 |
| eg: | Sample Vol | 3 µL |
| | Reagent Vol | 150 µL |
| Incubation Time | | 300 Seconds |
| Reagent Blank Limits | | Low 0.0 AU |
| (520nm, 1cm light path) | | High 0.5 AU |
| Linearity | | 0.03-1.50 mmol/L (0.5-25.2 mg/dL) |
| Analytical Sensitivity | | 0.42 ΔA per mmol/L |
| (520nm, 1cm light path) | | 0.025ΔA per mg/dL |

**CALCULATIONS**
Results are calculated, usually automatically by the instrument, as follows:

$$\text{Uric Acid} = \frac{\text{Absorbance of Unknown}}{\text{Absorbance of Calibrator}} \times \text{Calibrator Value}$$

**Example:**
Absorbance of calibrator = 0.302
Absorbance of unknown = 0.071
Value of calibrator = 0.720 mmol/L (12.1 mg/dL)

$$\text{Uric Acid} = \frac{0.071}{0.302} \times 0.720 = 0.16 \text{ mmol/L}$$

$$\text{Uric Acid} = \frac{0.071}{0.302} \times 12.1 = 2.8 \text{ mg/dL}$$

**NOTES**
1. The reagent and sample volumes may be altered proportionally to accommodate different spectrophotometer requirements.
2. The color development is stable for 15 minutes.
3. Specimens with Uric Acid concentrations greater than 1.50 mmol/L (25.2 mg/dL) should be diluted with saline and reassayed. Multiply results by the dilution factor.

Thermo SCIENTIFIC

4. S.I. unit conversion factor: mmol/L x 16.8 = mg/dL.

## CALIBRATION

Calibration is required. An aqueous standard or serum based calibrator, with an assigned value traceable to a primary standard (eg NIST or IRMM) is recommended. Standards should not contain formaldehyde or enzyme inhibitors as preservatives. For calibration frequency on automated instruments, refer to the instrument manufacturers specifications.

However, calibration stability is contingent upon optimum instrument performance and the use of reagents which have been stored as recommended in the stability and storage section of this package insert. Recalibration is recommended at anytime if one of the following events occurs:-

- The lot number of reagent changes.
- Preventative maintenance is performed or a critical component is replaced.
- Control values have shifted or are out of range and a new vial of control does not rectify the problem.

## QUALITY CONTROL

To ensure adequate quality control, normal and abnormal control with assayed values should be run as unknown samples:-

- At least once per day or as established by the laboratory.
- When a new bottle of reagent is used.
- After preventative maintenance is performed or a critical component is replaced.
- With every calibration.

Control results falling outside the upper or lower limits of the established ranges indicate the assay may be out of control.

The following corrective actions are recommended in such situations:-

- Repeat the same controls.
- If repeated control results are outside the limits, prepare fresh control serum and repeat the test.
- If results are still out of control, recalibrate with fresh calibrator, then repeat the test.
- If results are still out of control, perform a calibration with freshly prepared reagent, then repeat the test.
- If results are still out of control, contact Technical Services or the local distributor.

## LIMITATIONS

1. Studies to determine the level of interference from haemoglobin, bilirubin (free and conjugated) and lipaemia were carried out. The following results were obtained:
   **Haemoglobin:** No interference from haemoglobin up to 424 mg/dL.
   **Free Bilirubin:** No interference from free bilirubin up to 212 µmol/L (12 mg/dL).
   **Conjugated Bilirubin:** No interference from conjugated bilirubin up to 212 µmol/L (12 mg/dL).
   **Lipaemia:** No interference from lipaemia, measured as absorbance at 630nm, up to 1.68 AU.
2. Young DS[8] has published a comprehensive list of drugs and substances which may interfere with this assay.

## EXPECTED VALUES [9]

| | | |
|---|---|---|
| Child: | 0.12 - 0.32 mmol/L | 2.0 - 5.0 mg/dL |
| Adult Male: | 0.21 - 0.42 mmol/L | 3.5 - 7.2 mg/dL |
| Adult Female: | 0.15 - 0.35 mmol/L | 2.6 - 6.0 mg/dL |
| Urine: | 1.48 - 4.43 mmol/day | 250 - 750 mg/day |

The quoted values should serve as a guide only. It is recommended that each Laboratory verify this range or derives a reference interval for the population it serves. [10]

## PERFORMANCE DATA

The following data was obtained using the Infinity Uric Acid Liquid Stable Reagent on a well maintained automated clinical chemistry analyzer. Users should establish product performance on their specific analyzer used.

## IMPRECISION

Imprecision was evaluated over a period of 20 days using two levels of commercial control and following the NCCLS EP5-T procedure. [11]

| | | LEVEL I | LEVEL II |
|---|---|---|---|
| Number of data points | | 80 | 80 |
| Mean (mmol/L / mg/dL ) | | 0.279 / 4.69 | 0.603 / 10.13 |
| **Within run:** | SD (mmol/L / mg/dL) | 0.007 / 0.12 | 0.009 / 0.15 |
| | CV (%) | 2.3 | 1.5 |
| **Total:** | SD (mmol/L / mg/dL) | 0.019 / 0.32 | 0.021 / 0.35 |
| | CV (%) | 6.8 | 3.4 |

## METHOD COMPARISON

Comparison studies were carried out using a similar commercially available reagent as a reference. Serum and urine samples were assayed in parallel and the results compared by least squares regression. The following statistics were obtained.

| | |
|---|---|
| **Serum:** | |
| Number of sample pairs | 60 |
| Range of sample results | 0.11-0.61 mmol/L (1.85-10.25 mg/dL) |
| Mean of reference method results | 0.315 mmol/L (5.29 mg/dL) |
| Mean of Uric Acid results | 0.336 mmol/L (5.65 mg/dL) |
| Slope | 0.931 |
| Intercept | 0.042 mmol/L (0.71 mg/dL) |
| Correlation coefficient | 0.987 |
| **Urine:** | |
| Number of sample pairs | 41 |
| Range of sample results | 0.48 - 11.7 mmol/L (8.0 - 196 mg/dL) |
| Mean of reference method results | 3.0 mmol/L (49.6 mg/dL) |
| Mean of Uric Acid results | 2.5 mmol/L (42.7 mg/dL) |
| Slope | 0.967 |
| Intercept | -0.32 mmol/L (-5.3 mg/dL) |
| Correlation coefficient | 0.990 |

## LINEARITY

When run as recommended the assay is linear between 0.03 and 1.50 mmol/L (0.5-25.2 mg/dL).

## ANALYTICAL SENSITIVITY

When run as recommended the sensitivity of this assay is 0.42 $\Delta$Abs per mmol/L or 0.025 $\Delta$Abs per mg/dL (1cm light path, 520nm).

## REFERENCES


1. Searcy R.L., Diagnostic Biochemistry. McGraw-Hill, New York, NY, 1969.
2. Henry R.J, Common C. and Winkelman J.W. (eds), Clinical Chemistry: Principles and Techniques. Harper & Row, Hagerstown, MD, 1974.
3. Balis M. E., Adv. Clin. Chem. 18(213) 1976.
4. Trivedi R., Berta E. and Rebar L., Clin. Chem. 22(1223), 1976.
5. Kabasakalian P. Kalliney S. and Wescott A. Clin. Chem. 19(522) 1973.
6. Trinder P., J. Clin. Pathol. 22(246) 1949.
7. Shephard MD, Mezzachi RD. Clin Biochem Revs 1983; 4:61-7.
8. Young DS. Effects of Drugs on Clinical Laboratory Tests. Third Edition. 1990; 3:360-370.
9. Tietz Textbook of Clinical Chemistry and Molecular Diagnosis (4th Ed.) Burtis, Ashwood & Bruns (Eds), Elsevier Saunders, 2005; 2301.
10. Wachtel M et al, Creation and Verification of Reference Intervals. Laboratory Medicine 1995; 26:593-7.
11. National Committee for Clinical Laboratory Standards. User evaluation of Precision Performance of Clinical Chemistry Devices. NCCLS; 1984, NCCLS Publication EP5-T.



Fisher Diagnostics
a division of Fisher Scientific Company, LLC
a part of Thermo Fisher Scientific Inc.
Middletown, VA 22645-1905 USA
Phone: 800-528-0494
540-869-3200
Fax: 540-869-8132

EC REP WMDE
Bergerweg 18
6085 AT Horn
The Netherlands


CE

JL840768-en (R1)


*© 2012 Thermo Fisher Scientific Inc. All rights reserved. Hitachi is a registered trademark of Roch[illegible] Diagnostics, Indianapolis, IN 46250. ILab 600 is a registered trademark of Instrumentation Laborator[illegible] Company, Lexington, MA 02421. All other trademarks are the property of Thermo Fisher Scientific Inc[illegible] and its subsidiaries.*


REF **Reorder Information**

| Catalogue No. | Configuration |
|---|---|
| TR24321 | 2 x 125 mL |

# Section C Original $^{1}$H-NMR spectral data

The data include urine samples from all 24 experimental subjects and all four interventions (576 samples), as well as 72 QC samples, yielding a total of 648 study samples. Data from four of the subjects were, however, removed, as explained in the main text. The spectral region between 0.5 and 10 ppm, excluding the region of the water peak (4.72–4.88 ppm), of the original $^{1}$H-NMR spectrum of each of the urine samples analysed was divided into 0.02-ppm equal-sized bins. This resulted in 467 bins containing spectral data. The original, raw spectral data matrix of all the samples analysed for this study are given as an electronic file in Excel format (S2_File.xlsx). Table A represents a small extract from this file.

**Table A. A small extract from the file containing the raw $^1$H-NMR spectral data.**

| | | | | | | | | | | |
|---|---|---|---|---|---|---|---|---|---|---|
| | | | | **Bin:** | **1** | **2** | **3** | **...** | **466** | **467** |
| | | | | **Bin no:** | **9.99** | **9.969999** | **9.95** | **...** | **0.53** | **0.51** |
| **Batch** | **Sample Name** | **Case** | **Treatment** | **Time** | | | | | | |
| 1 | QC1 | F | QC | | 144185.2 | 85175.12 | 97222.12 | | 162878.1 | 164512.9 |
| 1 | F41-1 | F | Vehicle | –1 | 252890.2 | 171823.4 | 163215.5 | | 384216.4 | 266010.8 |
| 1 | F410 | F | Vehicle | 0 | 151299.1 | 100954.3 | 86908.15 | | 262467 | 216768.2 |
| 1 | F411 | F | Vehicle | 1 | 79268.87 | 105579.2 | 95048.77 | | 76325.53 | 126937.3 |
| 1 | F412 | F | Vehicle | 2 | 84927.78 | 101751.3 | 127028.2 | | 77872.51 | 68765.72 |
| 1 | F413 | F | Vehicle | 3 | 74747.86 | 144056.7 | 47937.61 | | 91158.41 | 58079.11 |
| 1 | F414 | F | Vehicle | 4 | 67488.71 | 70024.98 | 68237.62 | | 103052 | 89226.37 |
| 1 | F32-1 | F | Alcohol | –1 | 140557.8 | 103222.9 | 98290.31 | | 297911.4 | 217084.5 |
| 1 | F320 | F | Alcohol | 0 | 186432.5 | 118033 | 86594.67 | | 224899.6 | 205953.7 |
| 1 | F321 | F | Alcohol | 1 | 138804.5 | 89211.63 | 82459.67 | | 137392 | 87124.63 |
| 1 | F322 | F | Alcohol | 2 | 74946.59 | 76699.26 | 70328.33 | | 125527.2 | 139514.5 |
| 1 | F323 | F | Alcohol | 3 | 70183.16 | 75978.23 | 71975.92 | | 90170.89 | 86251.59 |
| 1 | F324 | F | Alcohol | 4 | 170387.9 | 119688.5 | 133812.7 | | 125650 | 97010.52 |
| 1 | QC2 | F | QC | | 148854.6 | 107287.3 | 109676.4 | | 194299.3 | 116989.8 |
| 1 | F22-1 | F | NAD | –1 | 86274.71 | 155479 | 145948.6 | | 176155.9 | 165445.7 |
| 1 | F220 | F | NAD | 0 | 137788.9 | 91139.97 | 80084.29 | | 169180.1 | 168179.4 |
| 1 | F221 | F | NAD | 1 | 101979 | 64201.66 | 84790.44 | | 92116.46 | 112283.7 |
| 1 | F222 | F | NAD | 2 | 81776.94 | 62372.04 | 73383.53 | | 94053.74 | 71139.18 |
| 1 | F223 | F | NAD | 3 | 103830 | 99023.64 | 83557.65 | | 87499.72 | 105244.2 |
| 1 | F224 | F | NAD | 4 | 81614.46 | 71494.57 | 85544.25 | | 99941.91 | 85364.31 |
| 1 | F14-1 | F | NAD + Alcohol | –1 | 167690.9 | 147427.5 | 99771.65 | | 171520.8 | 176960.6 |
| 1 | F140 | F | NAD + Alcohol | 0 | 79105.6 | 75316.07 | 71888.47 | | 111565.1 | 74134.41 |
| 1 | F141 | F | NAD + Alcohol | 1 | 67927.37 | 97794.58 | 50990.82 | | 76890.9 | 114086.5 |
| 1 | F142 | F | NAD + Alcohol | 2 | 70637.47 | 54988.69 | 86134.48 | | 69929.7 | 94239.57 |

The complete raw data are given as an electronic file in Excel format and is attached online as part of the Supporting Information (see S2_File.xlsx).

# References

1. Dona AC, Jiménez B, Schäfer H, Humpfer E, Spraul M, Lewis MR, et al. Precision high-throughput proton NMR spectroscopy of human urine, serum, and plasma for large-scale metabolic phenotyping. Anal Chem. 2014; 86(19):9887–9894.

2. Ellinger JJ, Chylla RA, Ulrich EL, Markley JL. Databases and Software for NMR-based metabolomics. Curr Metabolomics. 2013; 1:28–40.

3. Krojanski HG, Lambert J, Gerikalan Y, Suter D, Hergenröder R. Microslot NMR probe for metabolomics studies. Anal Chem. 2008; 80:8668-8672.

4. Smilde AKM, Van der Werf M, Bijlsma S, Van der Werff-van der Vat BJC, Jellema RH. Fusion of mass spectrometry-based metabolomics data. Anal Chem. 2005; 77:6729–6736.

5. ThermoFisher Scientific. Infinity™ Uric Acid Liquid Stable Reagent. 2012:1–2. Available: https://www.thermofisher.com/order/catalog/product/TR24321.
